# Knowledge, attitudes and practices toward diseases related to water, hygiene and sanitation among inhabitants of informal settlements in French Guiana

**DOI:** 10.3389/fpubh.2026.1749333

**Published:** 2026-04-10

**Authors:** Pierre Mahé, Estelle Thomas, Astrid Van Melle, Sébastien Rabier, Loic Epelboin, Margot Oberlis

**Affiliations:** 1 Croix Rouge française, Cayenne, French Guiana; 2 Centre d'Investigation Clinique Antilles-Guyane, CIC Inserm 1424, Centre Hospitalier de Cayenne, Cayenne, French Guiana; 3 Unité des Maladies Infectieuses et Tropicales, Centre Hospitalier de Cayenne, Cayenne, French Guiana

**Keywords:** communicable diseases, French Guiana, health knowledge, attitudes, practice, sanitation, urban slums, water supply

## Abstract

**Introduction:**

Informal settlements along French Guiana's coastline are characterized by precarious living conditions and limited access to water, hygiene, and sanitation (WASH), increasing residents' vulnerability to infectious diseases. This study assessed knowledge, attitudes and practices (KAP) regarding WASH-related diseases to inform targeted health promotion interventions.

**Methods:**

A sequential explanatory mixed-methods cross-sectional study was conducted from May to July 2023 in four informal settlements in Cayenne, Macouria, Matoury and Remire-Montjoly. Quantitative data were collected through an interviewer-administered survey, and qualitative data were gathered via focus group discussions to explore and contextualize the survey findings. This sequential design allowed qualitative findings to explain patterns observed in the quantitative data and to provide insights into contextual factors influencing residents' behaviors.

**Results:**

A total of 364 residents participated (exceeding the initially calculated sample size of 332). Participants had a mean age of 39.9 years [±12.97], and 60% were women. Most participants (98%) were born abroad, mainly in Haiti (84%). Nearly half (46%) reported consuming non-potable water and only 5% treated it correctly with disinfectants. Knowledge of diarrheal diseases was high (93%), whereas awareness of vector-borne and zoonotic diseases was low (14% for dengue fever; 35% for leptospirosis). Eighty-one percent reported using both individual and collective preventive measures. Sociodemographic characteristics were not associated with KAP outcomes.

**Discussion and Conclusion:**

These findings highlight persistent gaps in access to safe drinking water and effective water treatment practices, as well as limited awareness of diarrheal, vector-borne, and zoonotic diseases. Together, these factors emphasize the need for targeted health promotion strategies, preventive interventions, and community-level education to reduce disease risks in informal settlements of French Guiana.

## 1 Introduction

French Guiana, a French overseas territory in northeastern South America, covers approximately 84,000 km^2^ and is largely covered by the Amazon rainforest. Its population, concentrated along the coast in the main cities of Cayenne, Saint-Laurent-du-Maroni and Kourou, was estimated at around 300,000 in 2023 [National Institute of Statistics and Economic Studies (INSEE), 2021] (1). The population is young and culturally diverse, shaped by migrations from neighboring countries, overseas territories and more recent arrivals from regions such as Central Asia, the Middle East and North Africa. In recent years, the territory has even recorded a negative migratory balance, with slightly more people emigrating than immigrating (2–5). This diverse demographic and ethnic composition contributes to the uniqueness of French Guiana's society while also posing challenges in public health, education and social integration (6). Confronted with high poverty levels-−53% below the poverty line and nearly 30% in extreme poverty in 2023 (7–9)—a substantial portion of the population lives in informal settlements characterized by substandard housing and limited access to water, sanitation and electricity (10–12). Despite their illegal status, these settlements continue to expand, and with a quarter of the population living in unsanitary conditions, residents face significant health risks due to overcrowding and poor sanitation (10). Residents of these neighborhoods are particularly vulnerable to diseases related to water, hygiene and sanitation (WASH), as well as to the epidemic waves that regularly affect the territory. French Guiana experienced a dengue epidemic, with more than 22,709 clinically suspected cases and 11,343 confirmed cases recorded between January 2023 and July 2024 (13, 14). Residents of informal settlements are especially at risk due to the high density of mosquito breeding sites and the challenges of protecting themselves effectively. This situation is further aggravated by limited access to health information and medical care, which represents a major challenge for these populations. Often geographically isolated and faced with barriers such as poor infrastructure, a shortage of medical personnel and a lack of awareness of available healthcare services, these factors collectively increase their vulnerability to epidemics and preventable diseases. Leptospirosis, a bacterial zoonosis transmitted through contact with the urine from infected rodents, was designated a notifiable disease in France in August 2023 and represents another major health threat (15). According to a recent study on the epidemiology of leptospirosis in French Guiana between 2016 and 2022, the annual incidence rate fluctuated between 11 and 21 cases per 100,000 inhabitants from 2020 to 2022, with approximately one-third of patients originating from informal settlements (16). The absence of proper waste management facilities exacerbates this problem, as uncontrolled dumping attracts rodents that spread the disease. Additionally, the lack of adequate sanitation infrastructure results in the disposal of wastewater into the environment and poor rainwater drainage, further facilitating disease transmission (17). Limited access to drinking water is another major concern for residents in these settlements, particularly regarding enteric diseases. Economic constraints are a key issue: free emergency water distribution points, set up during the COVID-19 pandemic, have largely been removed and replaced with a limited number of prepaid water stations. Access to these fountains requires purchasing a prepaid card, costing approximately €40 for the initial purchase and €7.40 for a 5 m3 refill. This challenge is further compounded by geographical constraints, including an insufficient number of fountains relative to neighborhood density and the distance required to reach them. As of 2023, an estimated 18,000 spontaneous housing units in French Guiana remained unconnected to water and sanitation networks (18). As a result, many residents are forced to consume non-potable water, thereby increasing their risk of enteric diseases. Depending on the territory, the incidence rate of these diseases can be particularly high, reaching up to 50% among populations supplied by defective systems or not connected to public drinking water networks (19). This situation is illustrated by the findings of an investigation conducted by the health surveillance and management unit, which analyzed clustered cases of typhoid fever recorded between 1995 and 2007. Over this period, 13 typhoid fever outbreaks were identified in French Guiana, accounting for a total of 80 cases distributed across small clusters ranging from 2 to 13 cases. In nearly all episodes, the affected populations were not served by public drinking water networks and relied on unsafe water sources, such as river water in isolated areas or well water in urban settings (20). Despite the well-documented burden of WASH-related diseases and structural deficiencies in informal settlements, little is known about how residents perceive these risks, what they know about disease prevention, and how this understanding translates into daily practices in the specific context of French Guiana. Existing studies in the territory have primarily focused on epidemiological surveillance, infrastructure gaps, or programmatic descriptions, with limited empirical evidence on community-level knowledge, attitudes, and practices (KAP) related to WASH-associated diseases. Moreover, while numerous KAP studies have been conducted in informal settlements in other low- and middle-income settings, particularly in Africa and Asia, their findings cannot be directly extrapolated to French Guiana due to its unique socio-political status as an overseas collectivity of France, its cultural heterogeneity, and its specific legal and institutional context (21, 22). This represents a critical gap in the literature, as the lack of context-specific KAP data limits the design of effective, culturally adapted, and sustainable public health interventions. In response to these challenges, including restricted access to health information due to geographical and social isolation and low health literacy, the WASH project was implemented in French Guiana from 2020 to 2022. Developed by the French Red Cross and its partners, this initiative aimed to improve living conditions for vulnerable populations. Launched in the context of the COVID-19 pandemic, the project promoted good hygiene and sanitation practices through the provision of equipment and awareness campaigns. It also facilitated the creation of neighborhood committees composed of local residents, empowering them to lead collective initiatives for community improvement. Building on the WASH project, the Mobile Environmental Health Team (MHET) initiative was introduced to continue raising awareness of hygiene, sanitation, and water issues at both individual and community levels. The MHET project also sought to strengthen neighborhood committees to foster collective awareness and promote local empowerment (23–25). Within this context, the present study was conducted alongside the MHET project to better understand residents' perceptions and practices related to WASH-associated diseases, rather than to serve as a baseline evaluation prior to intervention activities. However, beyond program implementation, there has been no systematic assessment of residents' KAP related to WASH-associated diseases in these informal settlements. Without such evidence, it remains unclear whether existing interventions adequately address residents' perceptions, behaviors, and priorities, or how they could be optimized to achieve sustained health impact. To effectively implement the MHET project and better understand residents' needs, it was essential to assess their knowledge, attitudes and practices (KAP) regarding the prevention of diseases related to water, hygiene, and sanitation in informal settlements. Accordingly, this study aims to provide a cross-sectional assessment of KAP in an intervention-exposed real-world setting, generating novel, context-specific evidence to inform targeted, community-based, and policy-relevant public health actions.

## 2 Materials and methods

### 2.1 Study design

A sequential explanatory mixed-methods cross-sectional study was conducted from May 9 to July 13, 2023, as part of the Mobile Environmental Health Team (MHET) project. It assessed knowledge, attitudes, and practices (KAP) among residents of four informal settlements in Cayenne, Macouria, Matoury, and Remire-Montjoly. The neighborhoods were selected to capture diverse exposure to WASH-related awareness activities, including individuals who had previously benefited from MHET interventions and those who had not. The study followed a sequential explanatory mixed-methods design, with an initial quantitative survey followed by qualitative focus groups. Trained health mediators and volunteers from the French Red Cross and Médecins du Monde recruited adult participants and administered the questionnaire in the respondents' native language. The focus groups explored themes emerging from the quantitative survey to further explain and contextualize the findings. Convenience sampling was used due to high population mobility and the absence of a complete resident listing, but this approach captured a diversity of socio-demographic characteristics and prior exposure to MHET interventions, thus providing a sample relatively representative of the neighborhoods studied.

### 2.2 Survey population and study area

**Figure 1:**
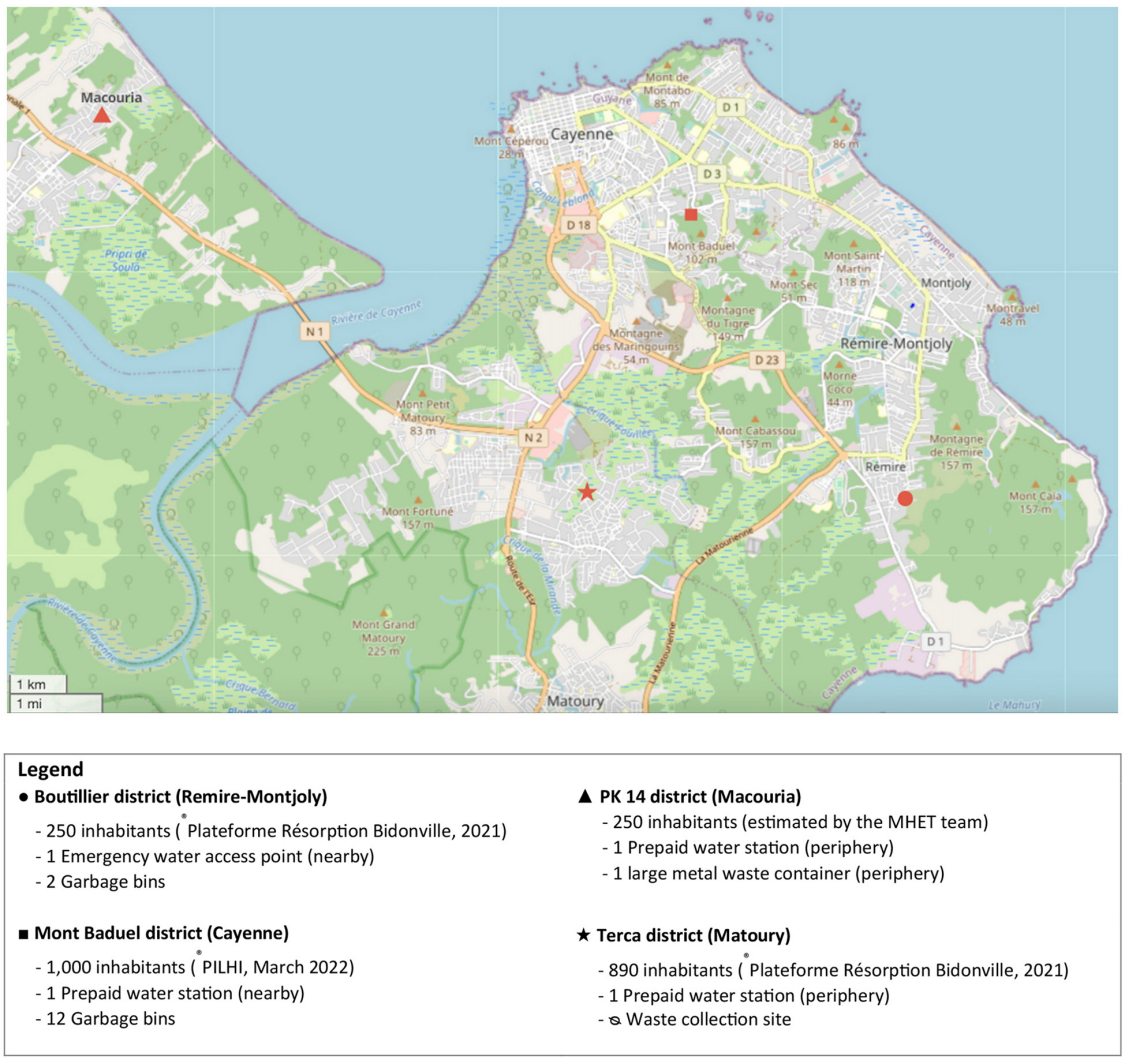
Map of investigated neighborhoods in and around Cayenne. Map data from OpenStreetMap, licensed under Open Data Commons Open Database License (38).

The survey targeted adults (≥18 years) living in four informal settlements: Boutillier (Rémire-Montjoly), Mont Baduel (Cayenne), PK 14 (Macouria), and Terca (Matoury). Neighborhood selection considered the project's area of intervention, the feasibility of future interventions, and differences in location and housing types (see Figure 1). Inclusion criteria were: age ≥18 years, residence in the neighborhood, and willingness to participate (oral non-opposition). Exclusion criteria included inability to participate due to language barriers not covered by the survey team.

### 2.3 Study sample calculation

Population estimates were obtained from the Résorption Bidonvilles platform, the Plan Intercommunal de Lutte contre l'Habitat Indigne (PILHI), and input from local stakeholders, including municipalities and associations, yielding a total of 2,390 residents in the targeted neighborhoods.

The following formula was used to determine the sample size for a proportional study:


$$\begin{array}{l} n=\frac{DEFF\;\times \;N\rho \left(1-\rho \right)}{\left(\frac{d^{2}}{Z_{1-\frac{\alpha }{2}}^{2}}\times \left(N-1\right)+\rho \left(1-\rho \right)\right)} \end{array}$$

Where:

- *N*: The number of residents in the selected neighborhoods
- *p*: The hypothetical percentage frequency of outcome factors in a population
- *d*: The precision, set at 5%
- DEFF: The design effect (set to 1)
- $Z_{1-\frac{\alpha }{2}}^{2}$: the critical value of the standard normal distribution

Given uncertainties in population data, convenience sampling was applied, and the formula served as a guide rather than implying representativeness. This approach ensured that the sample estimate approximated the population parameter as closely as possible, while recognizing that results were intended to provide an overview of KAP rather than generalize to all informal settlements. The final target sample size was set at 332 participants, proportionally distributed across neighborhoods. During data collection, 443 residents were approached; 65 refused participation, and 14 were excluded (7 did not live in targeted neighborhoods, 5 were under 18, and 2 withdrew). Consequently, 364 residents completed the survey, exceeding the target and yielding a response rate of 82.2%.

### 2.4 Data collection method and tools

#### 2.4.1 Quantitative survey

Face-to-face questionnaires were administered on tablets via the ODK Collect application (v2023.1). The 90-question survey covered: socio-demographics and prior interventions, WASH practices, and KAP related to diarrheal, vector-borne, and zoonotic diseases. The WASH section explored water access, collection, storage, treatment, sanitation, waste management, and hand hygiene. Participants reporting prior exposure to awareness or training activities were identified for comparative analyses. The questionnaire was pilot-tested by five health mediators and refined based on feedback.

#### 2.4.2 Qualitative survey

Focus groups were conducted with residents who completed the quantitative survey, including neighborhood committee members and other residents. Guides were based on initial survey results and addressed themes including health, environment, water access, waste management, and additional emerging issues. Sessions were moderated in participants' languages, including Haitian Creole. Each group consisted of 6–8 residents, with 29 participants across districts. Participation was voluntary and motivated by interest in the topics.

### 2.5 Data triangulation

To justify the use of a mixed-methods approach, Quantitative (KAP survey), qualitative (focus groups coded by neighborhood), and contextual data (water access, waste management, population estimates) were triangulated to provide a comprehensive view of WASH-related issues. See Table 1 for a summary of how these data sources were combined.

**Table 1:** Data triangulation showing integration of quantitative, qualitative and contextual data per neighborhood.

Neighborhood	Quantitative (KAP survey)	Qualitative (focus group discussions)	Contextual (environment)
Boutillier	*N* = 39 participants	R1-Bou, R2-Bou, R6-Bou	250 inhabitants; 1 emergency water access point (nearby); 2 Garbage bins
PK 14	*N* = 52 participants	R3-PK14, R5-PK14	250 inhabitants; 1 prepaid water station (periphery); 1 large metal waste container (periphery)
Mont Baduel	*N* = 168 participants	R4-MB	1 000 inhabitants; 1 prepaid water station (nearby); 12 Garbage bins
Terca	*N* = 105 participants	–	890 inhabitants; 1 prepaid water station (periphery); no waste collection site

The same KAP themes (WASH, diarrheal diseases, vector-borne diseases and zoonotic diseases) were explored in all neighborhoods. ≪R≫ = Resident, followed by a sequential number; ≪Bou≫ = Boutillier; ≪PK14≫ = PK 14; ≪MB≫ = Mont Baduel.

### 2.6 Ethical and data protection considerations

Residents were informed in advance about the survey dates and purpose. Before administering the questionnaire, interviewers introduced themselves and provided an information leaflet in the participant's native language, explaining the study and inclusion criteria. Participants were asked for oral non-opposition to the use of their anonymized responses for research purposes. For focus groups, written informed consent was obtained for audio recording. Recordings, which contained personal data (voices), were deleted 3 weeks after transcription, and only anonymized transcripts were used for analysis. In accordance with French and European regulations (Regulation 2016/679 General Data Protection Regulation) and due to the nature of the study (Research not involving human subjects—RnIPH—Articles 44.1 and 65.2 of the French Data Protection Act and Article L1110-12 of the French Public Health Code), submission to an Ethics Committee was not required.

### 2.7 Scoring system for quantitative variables

In the absence of validated Knowledge, Attitudes, and Practices (KAP) questionnaires for WASH-related diseases in informal settlements, an internal scoring system was developed for this study. It enabled descriptive and analytical interpretation of multiple-choice responses, providing an overview of residents' knowledge, attitudes, and practices. This questionnaire-specific tool was not intended as a validated measure. Thresholds for knowledge categories were defined *a priori* based on a review of previously used KAP questionnaires and public health recommendations, allowing differentiation between minimal, partial, and more comprehensive knowledge. Knowledge scores were assigned based on predefined correct responses for each thematic section (diarrheal, vector-borne, and zoonotic diseases) and classified into three categories: good, sufficient, or poor. Practices were similarly categorized according to compliance with recommended public health guidelines (e.g., safe water handling, hand hygiene). For disease-specific preventive behaviors, participants were categorized dichotomously as adopting at least one relevant action or none. This approach documented existing behaviors without judging effectiveness or cultural relevance. All scores were used in descriptive tables and statistical analyses. Detailed scoring criteria are provided in Supplementary Table S1.

### 2.8 Analysis plan

#### 2.8.1 Quantitative component

The questionnaire was designed on the KoboToolbox platform, and data were collected using ODK Collect (v2023.1) and entered into Microsoft Excel (v16.0). Quantitative analyses were conducted using R Studio (v4.3.0) and Excel. Multiple-choice responses were coded, grouped, and used to create composite variables, including KAP scores, as detailed in section 2.8. Categorical variables were expressed as frequencies and percentages, and continuous variables as means ± standard deviations. Associations between categorical variables were assessed with the chi-square (χ^2^) test. To examine how knowledge levels related to reported attitudes and practices, ordinal logistic regression was applied, as the dependent variable consisted of ordered categories. Models were unadjusted for sociodemographic characteristics, as bivariate analyses indicated no significant associations with KAP outcomes (age, sex, education, socio-professional category, neighborhood, country of birth, duration of residence). Results should therefore be interpreted as associations rather than causal effects. While the overall sample size was sufficient for descriptive analyses and assessing associations in the total population, statistical power for subgroup analyses (e.g., by neighborhood, age, or education) may be limited; non-significant findings in subgroups should be interpreted with caution. Results are reported as odds ratios (OR) with 95% confidence intervals, using a significance level of 0.05.

#### 2.8.2 Qualitative component

Focus groups were recorded and transcribed verbatim in Word. Using MAXQDA software (version 2022.6), the data were categorized by theme via axial coding of the transcripts. All quotations from focus group participants are labeled with a code indicating the participant and their neighborhood. “R” refers to “Resident”, followed by a sequential number for each citation, and a short abbreviation for the neighborhood (for example, R1-Bou refers to the first resident from Boutillier).

## 3 Results

### 3.1 Descriptive results

A total of 364 residents participated in the KAP survey (exceeding the initially calculated sample size of 332). Among them, 60% were women and 58% were over 35 years old (μ = 40 years; σ = 12.9). Despite 61% of participants having received a secondary education (middle school to high school level), a significant proportion were unemployed. Regarding length of residence, 41% had been living in their neighborhood for more than 5 years, while the average length of residence in French Guiana was approximately 10 years (σ = 10.6, see Table 2). The type of housing varied by neighborhood. In Mont Baduel, 68% of residents lived in tin structures, whereas in Boutillier, most residents lived in wooden dwellings. Overall, 61% of homes consisted of one to two rooms and households typically comprised of one to four individuals living under the same roof. Additionally, 94% of residents had access to toilets, with 57% using septic tanks and 25% using all-water tanks (see Supplementary Table S2). However, most of these toilets were homemade and substandard, exposing residents to unsanitary conditions. As one resident from the Boutillier neighborhood reported, she found herself in distress when heavy rainfall caused water levels to rise inside her home.

“There aren't even ten people who meet septic tank standards. [...] It can't even take a month to relieve yourself because it fills up too quickly. But you must spend a year with it, and when it rains, it comes back up. [...] One day, my house was flooded, it was as if all the sewage had come into my house. I cried and called for help. And it took my husband and I hours to clean up.” R1-Bou

**Table 2:** Description of socio-demographic characteristics of surveyed residents by studied neighborhood (May 2023).

Characteristic	Mont Baduel (Cayenne) *N* = 168	Boutillier (Remire-Montjoly) *N* = 39	PK 14 (Macouria) *N* = 52	Terca (Matoury) *N* = 105	Total *N* = 364
Age group				
< 25 years	10% [17]	8% [3]	13% [7]	10% [11]	10% [38]
25–35 years	41% [69]	31% [12]	31% [16]	17% [18]	32% [115]
35–45 years	30% [51]	33% [13]	37% [19]	25% [26]	30% [109]
45–55 years	13% [21]	23% [9]	13% [7]	20% [21]	16% [58]
55–65 years	4% [7]	5% [2]	4% [2]	13% [14]	7% [25]
>65 years	2% [3]	0%	2% [1]	14% [15]	5% [19]
Sex^a^				
Female	55% [92]	62% [24]	71% [37]	63% [66]	60% [219]
Male	45% [76]	38% [15]	29% [15]	37% [39]	40% [145]
Level of education				
Primary level	21% [35]	18% [7]	13% [7]	27% [28]	21% [77]
Secondary level	61% [103]	69% [27]	71% [37]	52% [55]	61% [222]
Tertiary level	11% [18]	0%	4% [2]	7% [7]	7% [27]
School level	7% [11]	10% [4]	12% [6]	13% [14]	10% [35]
Socio-professional category				
Unemployed, seeking work	59% [99]	51% [20]	42% [22]	25% [26]	46% [167]
Unemployed, not seeking work	27% [46]	38% [15]	33% [17]	21% [22]	27% [100]
Undeclared activity	8% [14]	8% [3]	21% [11]	22% [23]	14% [51]
Salaried	1% [1]	3% [1]	2% [1]	14% [15]	5% [18]
Retired	0%	0%	0%	12% [13]	4% [13]
Student	4% [6]	0%	2% [1]	3% [3]	3% [10]
Country of origin^b^				
Haiti	89% [149]	97% [38]	98% [51]	66% [69]	84% [307]
Brazil	0%	0%	0%	16% [17]	5% [17]
Western Sahara	10% [16]	0%	0%	0%	4% [16]
Suriname	0%	0%	2% [1]	6% [6]	2% [7]
French Guiana	1% [1]	0%	0%	6% [6]	2% [7]
Guyana	1% [1]	0%	0%	4% [4]	1% [5]
Dominican Republic	0%	3% [1]	0%	1% [1]	1% [2]
Saint Lucia	0%	0%	0%	2% [2]	1% [2]
Guinea	1% [1]	0%	0%	0%	1% [1]
Length of residence in French Guiana				
< 1 month	1% [2]	0%	0%	0%	1% [2]
1–6 months	9% [15]	0%	0%	0%	4% [15]
6–12 months	2% [3]	0%	2% [1]	0%	1% [4]
1–5 years	30% [50]	21% [8]	31% [16]	19% [18]	26% [92]
5–10 years	54% [90]	59% [23]	58% [30]	26% [25]	47% [168]
>10 years	4% [7]	21% [8]	10% [5]	56% [54]	21% [74]
Length of residence in the neighborhood				
< 1 month	4% [6]	0%	2% [1]	4% [4]	3% [11]
1–6 months	10% [17]	0%	4% [2]	5% [5]	7% [24]
6–12 months	6% [10]	0%	2% [1]	4% [4]	4% [15]
1–5 years	43% [72]	47% [17]	71% [37]	32% [33]	44% [159]
5–10 years	37% [61]	42% [15]	21% [11]	25% [26]	31% [113]
>10 years	1% [1]	11% [4]	0%	31% [32]	10% [37]

^a^Participants' sex was collected by self-report. Respondents indicated whether they were male or female. No other sex or gender categories were identified.

^b^Participants' country of birth was collected by self-report to assess whether cultural background could potentially influence the knowledge, attitudes, or practices under study.

In terms of access to drinking water, among the 364 participants, 50% reported relying on emergency water access points and prepaid water stations (PWS). In contrast, 45% reported using non-potable water sources, including well water and rainwater. Just over half of the participants reported treating their water before consumption (54%). Among the total study population, 47% reported using disinfectant products for water treatment. However, only 5% correctly applied the recommended dosage (Table 3). While practices regarding the time required for water treatment before consumption were generally adequate, storage practices for treated water varied. Focus group discussions revealed that many participants treated water as a precautionary measure, driven by a sense of caution rather than a thorough understanding of proper treatment methods. As one resident from Boutillier explained:

“For treatment, we use bleach or lemon juice, but we're not sure it's effective. We do it to relieve ourselves.” R2-Bou

**Table 3:** Description of water access and water treatment practices among residents of informal settlements (May 2023).

Characteristic	Count (*N* = 364)	Percentage (%)
Main source of water supply		
**Potable water**	**192**	**54**
Emergency water access points	112	31
Prepaid water stations	68	19
Purchase of bottled water	12	3
Individual houshold tap	3	1
**Non-potable water**	**168**	**45**
Individual well	74	20
Rainwater	38	10
Collective well	28	8
Surface water	25	7
**Does not know**	**4**	**1**
Water treatment		
Yes	195	54
No	169	46
Treatment with disinfectant products		
Good practices	18	5
Poor practices	152	42
Other treatment methods	25	7
Does not treat	169	46
Water treatment time before consumption		
	170	47
Good practices	135	37
Poor practices	35	10
Water storage time after treatment		
	170	47
Good practices	87	24
Poor practices	83	23

Furthermore, the disinfectant products observed were often unsuitable for water consumption, as they were intended for soil treatment use. At the neighborhood level, access to formal drinking water infrastructure was limited. Mont Baduel was the only neighborhood with both an emergency water access point and a prepaid water station (PWS). In contrast, each of the other neighborhoods had only one formal drinking water point: an emergency water access point in Boutillier and a prepaid water station in PK 14 and Terca. Despite the presence of these water points, focus group discussions indicated that the consumption of non-potable water remained common among residents. Overall, 29% of participants reported not using emergency water access points or PWS because of the distance to these facilities, and 20% cited financial constraints (see Supplementary Table S3). In addition, some residents reported consuming water they did not perceive as unsafe, such as spring wa ter in Mont Baduel, even for children, due to the absence of visible evidence of contamination. The remoteness of the water points also posed risks linked to road traffic, particularly at PK 14, where residents risked walking close to the road to obtain water at PK 16.

“Some people fetch water with a wheelbarrow, putting their container in it or going on foot. And that's dangerous! Four years ago, my cousin died on the road while fetching water. He'd put jerry cans on his scooter and was hit.” R3-PK14

The insecurity felt was also justified by the frequent presence of border police at the PWS. They carry out identity checks, which discourages illegal residents from going there during the day. This forced these people to collect water at night, where they might confront delinquents. This situation was reported by a resident of the Mont Baduel district:

“Where they put the water, it's really difficult for us. Because at six to nine in the evening, it's complicated. Last week, a gentleman was shot. They took his scooter and shot him twice. It wasn't even ten o'clock, so if the water was closer, it would be better for us.” R4-MB

Water collection, transport and storage practices were rated as good or sufficient. In terms of hand hygiene, 71% of participants said they washed their hands after using the toilet and 97% washed their hands with appropriate products (see Supplementary Tables S4, S5).

Regarding waste management, 80% of participants said they disposed of their waste at collection points, while 11% burned it (see Supplementary Table S6). However, some neighborhoods had no waste collection facilities, contradicting survey findings. Despite residents' awareness of the health risks associated with pollution and waste burning, a lack of motivation was conveyed during the focus groups. This led to interpersonal conflicts and a communication deficit.

For diarrheal diseases, 93% of those surveyed knew that they could be transmitted by feces. Eighty-eight percent had knowledge considered good or adequate about diarrhea transmission, while 13% had poor knowledge. The majority (88%) took a proactive approach to diarrheal diseases and 80% used preventive measures to avoid them (Table 4).

**Table 4:** Knowledge, attitudes and practices regarding diarrheal diseases among residents of informal settlements (May 2023).

Characteristic	Count (*N* = 364)	Percentage (%)
Feces: responsible for diseases		
Yes	338	93
No	24	7
Knowledge of diarrhea transmission		
Good knowledge	31	9
Sufficient knowledge	287	79
No knowledge	46	13
Attitudes toward diarrheal diseases		
Proactive approach	322	88
No action taken	42	12
Diarrhea prevention measures		
Proactive approach	292	80
No action taken	72	20

For vector-borne diseases, 98% of participants knew that mosquitoes are vectors of disease, but only 14% could identify dengue as such. Twenty-four percent had no specific knowledge and 6% mentioned other non-vector diseases (HIV, Typhoid …). Forty percent had no knowledge of the symptoms of dengue fever, but 79% had a proactive approach to its management. As for preventive measures, 90% had a proactive individual approach and 71% had a collective one (Table 5). Despite a generally satisfactory understanding of mosquito-borne diseases and their role as vectors, specific knowledge of mosquito proliferation is insufficient. Although the term “stagnant water” was mentioned in the focus groups, the factors cited to explain mosquito proliferation reveal certain gaps. None of the participants mentioned the presence of breeding grounds (or their concept) in clear water, but two main reasons were put forward to justify the presence of mosquitoes. The first relates to an environment deemed unsanitary due to the large amount of garbage in the vicinity. The second reason concerns water treatment, where residents observed larval breeding grounds appearing due to the products used to treat the water:

“I used to use bleach, but the problem is that it contains little bugs [references to worms], it's the chlorine that does it.” R5-PK14

**Table 5:** Knowledge, attitudes and practices regarding vector-borne diseases among residents of informal settlements (May 2023).

Characteristic	Count (*N* = 364)	Percentage (%)
Vectors: disease transmitters		
Yes	355	98
No	9	2
Knowledge of vector-borne diseases		
Dengue	50	14
Other (yellow fever, malaria, etc.)	115	32
Dengue and other	83	23
Incorrect understanding (HIV, typhoid, etc.)	22	6
No knowledge	86	24
Knowledge of dengue symptoms		
Good knowledge	98	27
Sufficient knowledge	120	33
No knowledge	146	40
Attitudes toward dengue		
Proactive approach	286	79
No action taken	78	21
Individual preventive measures		
Proactive approach	327	90
No action taken	37	10
Collective preventive measures		
Proactive approach	258	71
No action taken	106	29

For zoonotic diseases, 88% of participants knew that animals could transmit diseases. However, only 51% had heard of leptospirosis and 35% knew how it is transmitted. Ten percent had good knowledge of symptoms and 19% had sufficient knowledge. Forty-eight percent had a proactive approach to its management and 78% protected themselves individually while 87% used collective preventive measures (Table 6). Understanding of rodent-borne diseases was evident in the focus groups. Participants were aware of the risks and the unsanitary conditions in their neighborhoods and attributed the presence of rodents to the accumulation of garbage and the circulation of sewage.

“Where we live, if we don't keep our environment clean, we can catch a number of diseases. Rats can come by and do their business, and that can make us sick, and then we can get infections.” R6-Bou

**Table 6:** Knowledge, attitudes and practices regarding zoonotic diseases among residents of informal settlements (May 2023).

Characteristic	Count (*N* = 364)	Percentage (%)
Animals: disease transmitters		
Yes	319	88
No	45	12
Knowledge of leptospirosis		
Yes	185	51
No	179	49
Knowledge of leptospirosis transmission		
Yes	126	35
No	238	65
Knowledge of leptospirosis symptoms		
Good knowledge	35	10
Sufficient knowledge	69	19
No knowledge	260	71
Attitudes toward leptospirosis		
Proactive approach	175	48
No action taken	189	52
Individual preventive measures		
Proactive approach	284	78
No action taken	80	22
Collective preventive measures		
Proactive approach	315	87
No action taken	49	13

### 3.2 Analytical results

A bivariate analysis was carried out to study the association between socio-demographic characteristics and the KAP studied. The results showed that socio-demographic characteristics (neighborhood, age, gender, level of education, socio-professional category, country of birth, length of residence in French Guiana and in the neighborhood) were not significantly associated with water treatment, collection, transport, storage and hand hygiene practices. Similarly, they were not significantly associated with knowledge, attitudes and practices regarding diarrheal, vector-borne and zoonotic diseases. Detailed multivariable analyses supporting these findings are reported in Supplementary Tables S1.1–S5.6.

An ordinal logistic regression analysis then related knowledge of water-related diseases (diarrheal, vector-borne and zoonotic diseases) to associated attitudes and practices. This analysis found that higher knowledge levels were significantly associated with more appropriate attitudes and practices for most of the KAP studied (Table 7). Due to the cross-sectional design, these associations should not be interpreted as evidence of a causal relationship.

**Table 7:** Associations between knowledge levels and reported attitudes and practices regarding water-related diseases among surveyed residents (May 2023).

Characteristic	Diarrheal diseases					Dengue					Leptospirosis				
	***n*** **/** *N* ^*^	**%** ^**^	**OR**	**95% CI**	***p*** **-value**	***n*** **/** *N* ^*^	**%** ^**^	**OR**	**95% CI**	***p*** **-value**	***n*** **/** *N* ^*^	**%** ^**^	**OR**	**95% CI**	***p*** **-value**
Knowledge of symptoms															
Good knowledge	–	–	–	–	–	98/364	27	–	–		35/364	10	–	–	
Sufficient knowledge	–	–	–	–	–	120/364	33	0.29	[0.17–0.48]	< 0.001	69/364	19	0.51	[0.15–1.44]	0.2
No knowledge	–	–	–	–	–	146/364	40	0.16	[0.09–0.27]	< 0.001	260/364	71	0.20	[0.06–0.55]	0.003
Management attitudes															
Proactive approach	322/364	88	–	–		286/364	79	–	–		175/364	48	–	–	
Nothing	42/364	12	0.32	[0.15–0.71]	0.005	78/364	21	0.55	[0.33–0.92]	0.022	189/364	52	0.02	[0.01–0.05]	< 0.001
Individual preventive measures															
Proactive approach	292/364	80	–	–		327/364	90	–	–		284/364	78	–	–	
Nothing	72/364	20	0.11	[0.06–0.21]	< 0.001	37/364	10	0.76	[0.40–1.44]	0.4	80/364	22	1.19	[0.43–3.34]	0.7
Collective preventive measures															
Proactive approach	–	–	–	–	–	258/364	71	–	–		315/364	87	–	–	
Nothing	–	–	–	–	–	106/364	29	0.63	[0.40–0.97]	0.036	49/364	13	0.82	[0.19–3.51]	0.8

Method of analysis: ordinal logistic regression.

^*^n/N = number of participants who selected this response modality / total number of participants.

^**^% = percentage of participants who selected this response modality.

## 4 Discussion

To our knowledge, this is the first study investigating knowledge, attitudes, and practices (KAP) related to WASH-related diseases among residents of informal settlements in French Guiana. This survey provided an overview of KAP among residents living in different neighborhoods with varying contexts, particularly regarding their living environments. While some neighborhoods share similarities, each has its own specific context, making it difficult to generalize findings to all informal settlements in the CACL area. Nevertheless, the survey aimed to be as representative as possible by including neighborhoods of different typologies. Although numerous studies have examined KAP regarding water-related diseases, to date, we are not aware of any survey specifically addressing KAP in informal settlements, and even less so in French Guiana. Existing KAP studies focus mostly on displaced populations or villages in very different contexts, making direct comparisons challenging. However, some studies have addressed similar topics, particularly regarding water, hygiene, and sanitation. For example, a survey conducted by the CRf (Mobile Social Team) and the Regional Health Agency of Mayotte among informal settlements (26) reported comparable or even more pronounced results: difficulties in accessing water (87% of participants cited the distance to the BFM), waste management issues (57% of residents dispose of waste in open spaces, and one-third burn their waste), largely due to the lack of formal waste collection systems. In French Guiana, other surveys examining access to water, hygiene, and sanitation reveal practices similar to those observed in our study. A 2021 study (27) found that waste management relies primarily on collective bins provided by the municipalities, and that most households treat their water with chlorine or bleach, although knowledge regarding proper usage remains limited. These findings highlight persistent issues: insufficient sanitation, limited access to safe drinking water, and inadequate waste collection (28–30). Taken together, these results underscore the urgent need for municipalities and overseas departments and regions (DROMs) to address the needs of populations living in precarious conditions and highlight the importance of the present survey. KAP related to domestic water treatment require improvement. While certain practices, such as water collection, transport, and storage, are generally satisfactory, further efforts are needed to enhance awareness and train residents in optimal water treatment practices. This observation is consistent with findings from several studies in similar contexts, particularly in sub-Saharan Africa, where domestic water management remains marked by treatment deficiencies despite basic knowledge of health risks (31). The results indicate that although residents' knowledge is limited, preventive measures against water-related diseases are widely followed. While the study design does not allow for causal attribution, these behaviors highlight the relevance of ongoing interventions and encourage their continuation and reinforcement. This observation supports the value of health mediation and awareness activities, while emphasizing the need for further efforts to improve knowledge and practices, particularly regarding household water treatment. The survey further demonstrated that higher knowledge levels were associated with more appropriate attitudes and practices. Due to the cross-sectional design, it is not possible to infer causality, and these findings should be interpreted as associations rather than evidence that knowledge directly causes better practices. In addition, data collection was conducted over a limited period (May–July 2023), corresponding to the major rainy season in French Guiana. While this timing allowed observation of WASH conditions during a period of heightened environmental exposure (e.g., flooding, increased mosquito proliferation), the cross-sectional design does not allow assessment of seasonal variations in practices or disease awareness across different climatic periods. Studies conducted in Burkina Faso and Senegal have similarly shown that improved knowledge is directly linked to better water management and a reduced risk of waterborne diseases (32). The survey also enabled residents to express their concerns and needs. The primary issue raised concerned access to water. Residents clearly expressed a desire for closer proximity to water access points, as well as support for household water treatment. Improving access to water is particularly urgent in contexts such as Mayotte, where the cholera outbreak in June 2024 revealed the vulnerability of populations living in informal settlements to precarious hygiene conditions. This outbreak, which reported 221 cases, including 199 locally acquired, illustrates the severe health risks associated with the absence of safe drinking water and inadequate sanitation (33–35). Similar situations have been observed in French Guiana, where hygiene conditions and access to water remain problematic despite ongoing improvement efforts. These events emphasize the urgent need to strengthen drinking water and sanitation infrastructure to prevent future public health crises. Another major concern raised by residents relates to waste management. They stressed the need for greater awareness of proper waste disposal within neighborhoods, as well as the development and improvement of pre-collection facilities. This concern aligns with findings from numerous international studies, which identify waste management as a critical factor in preventing water-related diseases (36, 37). Finally, residents expressed an urgent need for improved access to health information. The findings suggest that awareness campaigns, support for neighborhood committees, and advocacy for the right to health are essential to promote healthier behaviors and enhance community resilience to health risks. A particularly concerning issue highlighted by this analysis is the tension between health authorities and local political leaders. Health authorities recognize the importance of improving access to safe drinking water, strengthening sanitation infrastructure, and supporting waste management to mitigate public health risks. However, political authorities are often reluctant to invest in informal settlements, fearing that such actions may be perceived as legitimizing these settlements and thus encouraging unregulated expansion. This dilemma underscores the need for a collaborative approach between political and health authorities to ensure that necessary investments are made while maintaining social balance and avoiding stigmatization. It should be noted that some participants had previously benefited from awareness-raising or training activities implemented by the MHET project, while others had no prior exposure. This design allowed for comparisons between exposed and unexposed participants, providing insights into both baseline KAP and potential early effects of interventions. However, some reported behaviors may reflect a social desirability bias, meaning that participants sometimes provided responses they perceived as socially acceptable or aligned with the interviewer's expectations, particularly for sensitive topics such as waste disposal or water treatment. While the presence of health mediators could have contributed to this tendency, this bias is not solely attributable to the interviewers, but rather represents a common challenge in survey-based studies assessing self-reported practices. This limitation should be taken into account when interpreting the results, particularly for behaviors that were difficult to verify through direct observation. Importantly, although the present study is discussed as a needs assessment, it was conducted in a context where environmental health interventions were already ongoing through the MHET project. The objective was therefore not to assess needs in the absence of interventions, but rather to document existing knowledge, attitudes, and practices in order to adapt, strengthen and complement ongoing actions and to identify unmet needs that are not sufficiently addressed by current interventions (for example, household-level water treatment practices). In this sense, the study can be considered a formative assessment, intended to inform the improvement and orientation of both current and future interventions. In addition, due to limited information on population numbers and the reliability of census data in informal settlements, a convenience sampling approach was adopted as the most feasible method. Although statistical analyses were conducted as if the sample were probabilistic, the results are intended to provide a snapshot of selected neighborhoods rather than to generalize to all informal settlements in the region. These findings offer an overview of local KAP and may provide useful insights for similar contexts, while acknowledging that each neighborhood has its own specific characteristics. Despite this, the survey still captures a broad spectrum of experiences and behaviors across neighborhoods, reflecting both populations reached and not yet reached by interventions, and the findings remain valuable for informing future public health actions.

## 5 Conclusion

This assessment of the WASH situation in the surveyed neighborhoods highlights the inadequacy of existing measures in preventing the health risks to which residents are exposed. The findings indicate that, despite ongoing efforts, significant gaps remain in the management of water, waste and WASH-related KAPs, as well as in the prevention of water-related diseases. Importantly, this assessment was conducted in parallel with ongoing MHET interventions and its purpose was to provide a formative evaluation to guide the improvement and adaptation of ongoing activities, as well as the development of complementary interventions such as household water treatment training.

This situation underscores the need to sustain and strengthen interventions under the MHET project, particularly in the field of health mediation. Health mediation plays a crucial role in improving knowledge, attitudes and practices (KAPs), especially in protecting individuals from water-related diseases. Beyond simply disseminating information, health mediation provides guidance, facilitates access to health services and rights, and enhances health literacy. By doing so, it may empower individuals to navigate the healthcare system more effectively, which may be associated with increased care-seeking behaviors and potentially better prevention and treatment adherence.

Indeed, the survey results indicate that greater knowledge is associated with more appropriate health behaviors, highlighting the importance of continuous awareness efforts to support household water management and disease prevention. While the study cannot directly link these behaviors to intervention effectiveness, the observed preventive practices may reflect the potential impact of health mediation and awareness activities.

Beyond the educational aspect, a cross-functional approach is imperative to enhance the sustainability of informal settlements and reduce health risks. This requires the implementation of innovative and tailored solutions, such as closer prepaid water stations to improve water access and a revision of waste pre-collection systems to better address residents' needs. Joint advocacy between health authorities, local policymakers and communities is necessary to adapt and scale up these existing structures, ensuring that they effectively meet residents' expectations. Moreover, this integrated approach will foster stronger collaboration between various stakeholders and institutions, facilitating enhanced coordination and more effective health monitoring. The Mayotte case, particularly the cholera outbreak in early 2024, illustrates the severe consequences of inadequate access to clean water and sanitation, reinforcing the urgent need for a systemic and collaborative response. Such actions will not only improve living conditions but also reduce exposure to unhealthy environments, prevent epidemics and strengthen community resilience in the face of health risks.

## Data Availability

The original contributions presented in the study are included in the article/Supplementary material, further inquiries can be directed to the corresponding author.
