# Supplementary material for: Knowledge, attitudes and practices toward diseases related to water, hygiene and sanitation among inhabitants of informal settlements in French Guiana
Source: Front Public Health. 2026 Apr 10;14:1749333. doi: 10.3389/fpubh.2026.1749333 (PMC13106344; doi:10.3389/fpubh.2026.1749333)
Supplement: Supplementary file 1 [file Supplementary_file_1.docx]

*Supplementary Material*

*Table S1. Scoring system used for quantitative KAP variables*

| **Variable** | **Context** | **Scoring criteria** | **Score attribution** | **Final categorization** |
| --- | --- | --- | --- | --- |
| See in Table 9. Knowledge, attitudes and practices regarding diarrheal diseases among residents of informal settlements (May 2023) | | | | |
| **Knowledge of diarrhea transmission** | Knowledge of transmission routes | Correct responses: contaminated water; contaminated/spoiled food; poor hand hygiene; contact with infected person; validated “other” | +1 per correct response | 0 : No knowledge ; 1-2 : Sufficient knowledge ; ≥ 3 : Good knowledge |
| **Attitudes toward diarrheal diseases** | Attitudes : Responses to illness | Medication use; traditional medicine; dietary adaptation; increased water intake; healthcare consultation; validated “other” | ≥1 action = proactive | Proactive approach / No action taken |
| **Diarrhea prevention measures** | Practices : Preventive measures | Water treatment; toilet use; handwashing with soap; food hygiene; household cleanliness; water protection | ≥1 action = proactive | Proactive approach / No action taken |
| See in Table 10. Knowledge, attitudes and practices regarding vector-borne diseases among residents of informal settlements (May 2023) | | | | |
| **Knowledge of dengue symptoms** | Knowledge of symptoms | Fever (+2); headache (+2); joint/muscle pain (+2); chills (+1); fatigue (+1); digestive symptoms (+1); others (0/+1) | Sum of weighted points | 0 : No knowledge ; 1-4 : Sufficient knowledge ; ≥ 5 : Good knowledge |
| **Attitudes toward dengue** | Attitudes : Response to illness | Medication; traditional medicine; dietary change; rest; hydration; healthcare consultation | ≥1 action = proactive | Proactive approach / No action taken |
| **Individual preventive measures** | Practices : Individual prevention | Mosquito net; repellents; coils; electric diffusers; long clothing; smoke | ≥1 action = proactive | Proactive approach / No action taken |
| **Collective preventive measures** | Practices : Environmental prevention | Removing standing water; covering containers; cleaning surroundings; waste removal | ≥1 action = proactive | Proactive approach / No action taken |
| See in Table 11. Knowledge, attitudes and practices regarding zoonotic diseases among residents of informal settlements (May 2023) | | | | |
| **Knowledge of leptospirosis symptoms** | Knowledge of symptoms | Fever (+2); headache (+2); muscle/joint pain (+2); chills (+1); digestive symptoms (+1); validated “other” | Sum of weighted points | 0 : No knowledge ; 1-4 : Sufficient knowledge ; ≥ 5 : Good knowledge |
| **Attitudes towards leptospirosis** | Attitudes : Response to illness | Medication; traditional medicine; dietary change; rest; hydration; healthcare consultation | ≥1 action = proactive | Proactive approach / No action taken |
| **Individual preventive measures** | Practices : Individual prevention | Food protection; safe water storage; protective footwear; wound protection; hygiene | ≥1 action = proactive | Proactive approach / No action taken |
| **Collective preventive measures** | Practices : Environmental prevention | Proper waste disposal; rodent control; environmental cleaning; avoiding stagnant water | ≥1 action = proactive | Proactive approach / No action |
| See in Table 4. Description of water access and water treatment practices among residents of informal settlements (May 2023) | | | | |
| **Treatment with disinfectant products** | Treatment method | Correct product and dosage | Correct = good practice | Good / Poor practices / Other treatment methods |
| **Water treatment time before consumption** | Treatment time before consumption | Adequate waiting time respected | 1 point = good practices | Good / Poor practices |
| **Water storage time after treatment** | Treatment time after treatment | Storage duration compatible with safe consumption | 1 point = good practices | Good / Poor practices |
| See in Table 6. Water collection, transport, and storage practices among surveyed residents of informal settlements (May 2023) | | | | |
| **Type of container used** | Water collection practices | Plastic bottle and/or glass bottle = 1 point | 1 point = or > good practices | Good / Poor practices |
| **Frequency of container washing** | Frequency of container cleaning | Before each use and/or at least once per week = 1 point | 1 point = or > good practices | Good / Poor practices |
| **Washing of storage containers** | Method for cleaning storage containers | Water and soap and/or disinfectant product (e.g., bleach) = 1 point | 1 point = or > good practices | Good / Poor practices |
| **Closing of containers during transport** | Container closure during transport | Always or sometimes covered = 1 point | 1 point = or > good practices | Good / Poor practices |
| **Closing of containers during storage** | Container closure during storage | Always or sometimes covered = 1 point | 1 point = or > good practices | Good / Poor practices |
| **Water storage at home** | Storage conditions | Location, elevation, protection (point-based system) | 0 point = poor ; 1 point = sufficient ; 2-5 points = good | Poor practices / Adequate practices / Good practices |
| See in Table 7. Hand hygiene practices among residents of informal settlements (May 2023) | | | | |
| **Hand hygiene practices** | Handwashing practices | Water and soap; alcohol-based gel; validated alternative | Correct method = good practice | Good / Poor practices |

#### Table S2. Description of the housing conditions of surveyed individuals by studied neighborhood (May 2023)

|  | Mont Baduel (Cayenne)  N = 168 | Boutillier (Remire-Montjoly)  N = 39 | PK 14 (Macouria)  N = 52 | Terca (Matoury)  N = 105 | Total  N = 364 |
| --- | --- | --- | --- | --- | --- |
| **Type of housing** |  |  |  |  |  |
| Sheet metal house  Wooden house  Concret house  Wooden house on stilts | 68% [114]  30% [51]  2% [3]  0 | 26% [10]  67% [26]  8% [3]  0 | 37% [19]  31% [16]  33% [17]  0 | 10% [11]  20% [21]  60% [63]  10% [10] | 42% [154]  31% [114]  24% [86]  3% [10] |
| **Number of rooms** |  |  |  |  |  |
| 1 room  2 rooms  3 to 4 rooms  5 to 10 rooms | 31% [52]  44% [74]  24% [41]  1% [1] | 32% [12]  34% [13]  32% [12]  3% [1] | 8% [4]  40% [21]  46% [24]  6% [3] | 14% [15]  28% [29]  47% [49]  11% [12] | 23% [83]  38% [137]  35% [126]  5% [17] |
| **Household size (people living in the home)** | | | | |  |
| 1 to 4 people  5 to 8 people  > 8 | 84% [141]  4% [6]  13% [21] | 87% [33]  0  13% [5] | 84% [43]  0  16% [8] | 70% [67]  5% [5]  25% [24] | 80% [284]  16% [58]  3% [11] |
| **Presence of toilet** | | | | |  |
| Yes  No | 94% [158]  6% [10] | 92% [36]  8% [3] | 96% [50]  4% [2] | 93% [98]  7% [7] | 94% [342]  6% [22] |
| **Type of toilet** | | | | |  |
| Septic tank  All-waters tank  Pit latrine  Dry toilet | 64% [107]  16% [27]  11% [18]  0 | 51% [20]  36% [14]  5% [2]  0 | 46% [24]  31% [16]  15% [8]  2% [1] | 51% [54]  30% [31]  10% [11]  0 | 57% [205]  25% [88]  12% [39]  1% [1] |

#### **Table S3.** Description of water supply via emergency access standpipes or public taps and reasons for non-use among residents of informal settlements (May 2023) ^[[1]](#footnote-1)^

3 Since these questions are multiple-choice, it is normal for the numbers to exceed the population of the neighborhood.

|  | Mont Baduel (Cayenne)  N = 168 | Boutillier (Remire-Montjoly)  N = 39 | PK 14 (Macouria)  N = 52 | Terca (Matoury)  N = 105 | Total  N = 364 |
| --- | --- | --- | --- | --- | --- |
| **Water supply via *Emergency water access points and Prepaid water stations*** | | | | | |
| Emergency water access points  Prepaid water stations | 45% [75]  17% [29] | 95% [37]  0 | 0  29% [15] | 0  23% [24] | 31% [112]  19% [68] |
| **Reasons for non-use of *Emergency water access points and Prepaid water stations*** | | | | | |
| Not available  Distance  Too expensive  Transportation issue to reach SGDE  Feasibility  Administrative  Not working  Security concerns  Border Police  Other  Don’t know | 10% [17]  19% [32]  27% [45]  3% [5]  1% [1]  4% [7]  1% [1]  10% [16]  4% [7]  4% [7]  7% [11] | 0  13% [5]  0  0  0  0  0  0  0  0  5% [2] | 15% [8]  44% [23]  21% [11]  2% [1]  0  2% [1]  0  0  0  0  10% [6] | 12% [13]  43% [45]  17% [18]  6% [6]  1% [1]  3% [3]  0  0  0  6% [6]  9% [9] | 10% [38]  29% [105]  20% [74]  3% [12]  1% [2]  3% [11]  1% [1]  4% [16]  2% [7]  4% [13]  8% [28] |

#### **Table S4.** Water collection, transport, and storage practices among surveyed residents of informal settlements (May 2023) ^[[2]](#footnote-2)^

4 The fact that some variables do not reach 100% of responses in this table is due to the fact that 1% of the residents [2 surveyed] have a faucet connected to a drinking water network.

|  | Count (N = 364) | Percentage (%) |
| --- | --- | --- |
| **Type of container used** | | |
| Good practices  Poor practices | 234  130 | 64  36 |
| **Frequency of container washing** | | |
| Good practices  Poor practices  Individual tap  No answer | 328  20  2  14 | 90  5  1  4 |
| **Washing of storage containers** | | |
| Good practices  Poor practices  No answer | 330  16  18 | 91  4  5 |
| **Closing of containers during transport** | | |
| Good practices  Poor practices  No answer | 311  39  12 | 85  11  3 |
| **Closing of containers during storage** | | |
| Good practices  Poor practices  No answer | 322  27  13 | 88  7  4 |
| **Water storage at home** |  |  |
| Good practices  Adequate practices  Poor practices | 89  249  26 | 24  68  7 |

#### **Table S5.** Hand hygiene practices among residents of informal settlements (May 2023)

|  | Count (N = 364) | Percentage (%) |
| --- | --- | --- |
| **Water point near the toilet** | | |
| Yes  No | 253  111 | 70  30 |
| **Hand hygiene after using the toilet** | | |
| Washes hands after using the toilet  Does not wash hands after using the toilet | 260  104 | 71  29 |
| **Hand hygiene practices** | | |
| Good practices  Poor practices  No answer | 353  8  3 | 97  2  1 |
| **Perception of hand hygiene** | | |
| Very good  Good  Not sure  No answer | 265  81  12  6 | 73  22  3  2 |

#### **Table S6.** Opinions and practices regarding waste management among residents of informal settlements (May 2023) ^[[3]](#footnote-3)^

5 Since these questions are multiple-choice, it is normal for the numbers to exceed the total population.

|  | Count (N = 364) | Percentage (%) |
| --- | --- | --- |
| **Opinion on waste management** | | |
| Better image of the neighborhood  Environment  Diseases  Unpleasant odors  Flooding  Toxic fumes  Presence of mosquitoes  Presence of rats  Presence of flies  Injuries  Presence of snakes  Does not know | 216  139  113  101  84  45  33  29  23  7  1  4 | 59  38  31  28  23  12  9  8  6  2  0.3  1 |
| **Waster management method** | | |
| Collection site  Burned  Illegal dumping site  Nature  Buried  Nothing | 293  39  27  21  2  2 | 80  11  7  6  0.6  0.6 |
| **Opinion on the waste collection site** | | |
| Satisfactory  Far away  Undersized  Not used  Insecurity  Trash bins damaged  Absent  Other  Does not know  No answer | 86  135  87  65  17  17  15  10  8  3 | 24  37  24  18  5  5  4  3  2  1 |

1. Supplementary Material – Detailed bivariate and multivariate analyses of knowledge, attitudes and practices related to diarrheal diseases (May 2023)

This supplementary material presents the detailed bivariate and multivariate analyses of variables related to knowledge, attitudes and practices on diarrheal diseases, corresponding to Table 9 in the main manuscript.

The analyses suggest that sociodemographic factors do not meaningfully explain variations in knowledge, attitudes, or practices related to diarrhoeal diseases. Although age was statistically significant in two models, the associations were weak and inconsistent, precluding any firm conclusion.

- 1. **Variable:** **« Feces: responsible for diseases »**

*Table S1.1. Multivariate logistic regression (outcome:* Feces: responsible for diseases*)*

Legend: In the multivariate logistic regression model, age was significantly associated with the belief that one can catch diseases (OR = 0.95; 95% CI 0.90–0.996; p = 0.042), suggesting slightly lower odds with increasing age. Living in Boutillier (Remire) showed a trend toward higher odds compared to 30_Pieces (Cayenne) (OR = 3.08; p = 0.065). Other sociodemographic factors were not significantly associated. The education variable remained unstable due to small subgroup sizes.

| **Variable** | **OR** | **95% CI** | **p-value** |
| --- | --- | --- | --- |
| **Age (per year)** | 0.95 | 0.90–0.996 | 0.042 |
| **Neighborhood (ref = 30_Pieces)** |  |  |  |
| Boutillier (Remire) | 3.08 | 0.87–9.95 | 0.065 |
| PK14 (Macouria) | 2.02 | 0.62–6.17 | 0.220 |
| Terca (Matoury) | 0.63 | 0.14–2.30 | 0.506 |
| **Sex (ref = female)** |  |  |  |
| Male | 0.56 | 0.20–1.43 | 0.243 |
| **Education (ref = non-schooled)** |  |  |  |
| Schooled | 4.48 × 10⁶ | 4.96e-15–3.18e+142 | 0.988 |
| **Socio-professional category (ref = salaried)** |  |  |  |
| Other | 2.08 | 0.36–17.47 | 0.443 |
| Inactive | 0.77 | 0.17–5.53 | 0.759 |
| **Origin (ref = France)** |  |  |  |
| Others | 0.36 | 0.04–7.99 | 0.408 |

- 1. **Variable: « Knowledge of diarrhea transmission »**

*Table S1.2. Ordinal logistic regression (outcome: knowledge of diarrhea transmission)*

Legend: In the ordinal logistic regression model, none of the sociodemographic characteristics were significantly associated with knowledge of diarrhoea transmission. There was a non-significant trend for men to have lower knowledge compared to women (OR = 0.62; 95% CI 0.36–1.06; p = 0.082).

| **Variable** | **OR** | **95% CI** | **p-value** |
| --- | --- | --- | --- |
| **Age (per year)** | 1.00 | 0.98–1.02 | 0.992 |
| **Neighborhood (ref = 30_Pieces)** |  |  |  |
| Boutillier (Remire) | 0.85 | 0.37–1.94 | 0.692 |
| PK14 (Macouria) | 0.85 | 0.39–1.83 | 0.672 |
| Terca (Matoury) | 1.09 | 0.55–2.16 | 0.813 |
| **Sex (ref = female)** |  |  |  |
| Male | 0.62 | 0.36–1.06 | 0.082 |
| **Education (ref = non-schooled)** |  |  |  |
| Schooled | 1.09 | 0.44–2.73 | 0.851 |
| **Socio-professional category (ref = salaried)** |  |  |  |
| Other | 0.76 | 0.24–2.42 | 0.648 |
| Inactive | 0.51 | 0.18–1.41 | 0.194 |
| **Origin (ref = France)** |  |  |  |
| Others | 1.11 | 0.16–7.53 | 0.917 |

- 1. **Variable:** **« Attitudes toward diarrheal diseases »**

*Table S1.3. Multivariate logistic regression (outcome:* Attitudes toward diarrheal diseases)

Legend: In the multivariable logistic regression model, most sociodemographic factors were not significantly associated with proactive diarrhoea management behaviours. However, being male was associated with lower odds of proactive behaviour (OR = 0.36; 95% CI 0.18–0.71), while belonging to the “Other” occupational category was associated with higher odds (OR = 6.09; 95% CI 1.47–31.63).

| **Variable** | **OR** | **95% CI** | **p-value** |
| --- | --- | --- | --- |
| **Age (continuous)** | 1.02 | 0.99–1.05 | 0.27 |
| **Neighborhood (ref = 30_Pieces)** |  |  |  |
| • Boutillier (Remire) | 0.82 | 0.29–2.66 | 0.71 |
| • PK14 (Macouria) | 1.31 | 0.45–4.80 | 0.65 |
| • Terca (Matoury) | 0.96 | 0.39–2.49 | 0.93 |
| **Sex (ref = female)** |  |  |  |
| • Male | 0.36 | 0.18–0.71 | 0.004 |
| **Education (ref = non-schooled)** |  |  |  |
| • Schooled | 0.33 | 0.02–1.77 | 0.30 |
| **Socio-professional category (ref = salaried)** |  |  |  |
| • Other | 6.09 | 1.47–31.63 | 0.018 |
| • Inactive | 2.30 | 0.79–6.20 | 0.11 |
| **Origin (ref = France)** |  |  |  |
| • Others | 0.75 | 0.04–5.52 | 0.81 |

- 1. **Variable:** **« Diarrhea prevention measures »**

*Table S1.4. Multivariate logistic regression (outcome:* Diarrhea prevention measures*)*

Legend: In multivariate logistic regression, only age was significantly associated with proactive behaviour to prevent diarrhoea (OR = 1.03; 95% CI 1.01–1.06; p = 0.019). None of the other sociodemographic variables were significantly associated with this attitude.

| **Variable** | **OR** | **95% CI** | **p-value** |
| --- | --- | --- | --- |
| **Age (continuous)** | 1.03 | 1.01–1.06 | 0.019 |
| **Neighborhood (ref = 30_Pieces)** |  |  |  |
| • Boutillier (Remire) | 2.08 | 0.75–7.36 | 0.199 |
| • PK14 (Macouria) | 0.92 | 0.42–2.15 | 0.832 |
| • Terca (Matoury) | 0.62 | 0.31–1.26 | 0.185 |
| **Sex (ref = female)** |  |  |  |
| • Male | 0.60 | 0.34–1.06 | 0.078 |
| **Education (ref = non-schooled)** |  |  |  |
| • Schooled | 0.76 | 0.21–2.21 | 0.639 |
| **Socio-professional category (ref = salaried)** |  |  |  |
| • Other | 1.56 | 0.40–5.88 | 0.510 |
| • Inactive | 0.59 | 0.18–1.60 | 0.334 |
| **Origin (ref = France)** |  |  |  |
| • Others | 0.89 | 0.11–5.01 | 0.898 |

2. Supplementary Material – Detailed bivariate and multivariate analyses of knowledge, attitudes and practices related to vector-borne diseases (May 2023)

This supplementary material presents the detailed bivariate and multivariate analyses of variables related to knowledge, attitudes and practices on vector-borne diseases, corresponding to Table 10 in the main manuscript.

The analyses suggest that sociodemographic factors do not meaningfully explain variations in knowledge, attitudes, or practices related to dengue and mosquito prevention. Although a few associations reached statistical significance, they were limited and often unstable due to small subgroup sizes, and most relationships were weak or inconsistent. Overall, no clear sociodemographic pattern emerged, indicating that other factors (e.g., personal experience, health communication, or community exposure) may better explain the observed differences in knowledge and behaviours.

**2.1. Variable: « Vectors: disease transmitters »**

*Table S2.1. Multivariate logistic regression (outcome:* Vectors: disease transmitters*)*

Legend: In the multivariate logistic regression model, sex was the only sociodemographic factor significantly associated with the belief that mosquitoes transmit diseases. Male participants had significantly lower odds of believing that mosquitoes are disease transmitters compared with females (OR = 0.14; 95% CI 0.02–0.63; p = 0.020). No significant associations were observed for age, neighborhood, education level, socio-professional category, or origin. Several variables, particularly education, occupational category, and origin, showed unstable estimates with extremely wide confidence intervals, reflecting very small subgroup sizes and quasi-separation.

| **Variable** | **OR** | **IC 95%** | **p-value** |
| --- | --- | --- | --- |
| Age (per year) | 1.07 | 0.99 – 1.17 | 0.122 |
| Quartier (ref = 30_Pieces) |  |  |  |
| • Boutillier (Remire) | 0.95 | 0.14 – 18.93 | 0.967 |
| • PK14 (Macouria) | 0.96 | 0.14 – 19.15 | 0.970 |
| • Terca (Matoury) | 1.35 | 0.18 – 28.07 | 0.798 |
| Sexe (ref = femme) |  |  |  |
| • Homme | 0.14 | 0.02 – 0.63 | 0.020 |
| Éducation (ref = non scolarisé) |  |  |  |
| • Scolarisé | 0.00 | NA – 2.78e+90 | 0.995 |
| CSP (ref = salarié) |  |  |  |
| • Autres | 2.57e+07 | 4.91e-75 – NA | 0.994 |
| • Inactifs | 0.97 | 0.05 – 6.89 | 0.977 |
| Origine (ref = France) |  |  |  |
| • Others | 0.00 | NA – 2.13e+214 | 0.998 |

**2.2. Variable: « Knowledge of vector-borne diseases »**

Model: Multinomial logistic regression (reference category: No knowledge)

*Table S2.2. Multinomial logistic regression (outcome:* Knowledge of vector-borne diseases*)*

Legend: In the multinomial logistic regression model adjusted for age, neighborhood, sex, education level, occupational category, and origin, no consistent or robust associations were observed between sociodemographic factors and knowledge of dengue transmission. Most odds ratios were close to unity and not statistically significant across outcome categories. The category “Origin: Other” showed very large odds ratios in some comparisons, particularly in the “Others” knowledge category, reflecting unstable estimates due to very small cell counts. These results should therefore be interpreted with caution. Overall, sociodemographic characteristics did not meaningfully explain variations in knowledge of dengue transmission in this population.

| **Category (reference = No knowledge)** | **Variable** | **OR** | **95% CI** | **p-value** |
| --- | --- | --- | --- | --- |
| **Others** | age (years) | 0.99 | 0.97–1.02 | 0.602 |
|  | Neighborhood: Boutillier (Rémire) | 0.47 | 0.19–1.21 | 0.117 |
|  | Neighborhood: PK14 (Macouria) | 1.23 | 0.54–2.79 | 0.627 |
|  | Neighborhood: Terca (Matoury) | 1.53 | 0.67–3.52 | 0.315 |
|  | Sex: male | 0.85 | 0.46–1.56 | 0.594 |
|  | Education: schooled | 2.33 | 0.79–6.90 | 0.127 |
|  | Occupational category: others | 1.81 | 0.39–8.51 | 0.452 |
|  | Occupational category: inactive | 2.25 | 0.56–9.08 | 0.256 |
|  | Origin: others | 423 | 144.82–1236.77 | <0.001 |
| **Dengue** | age (years) | 0.99 | 0.96–1.02 | 0.450 |
|  | Neighborhood: Boutillier (Rémire) | 1.00 | 0.95–1.06 | 0.264 |
|  | Neighborhood: PK14 (Macouria) | 1.01 | 0.97–1.06 | 0.489 |
|  | Neighborhood: Terca (Matoury) | 1.01 | 0.97–1.06 | 0.489 |
|  | Sex: male | 1.01 | 0.93–1.10 | 0.730 |
|  | Education: schooled | 1.00 | 0.97–1.03 | 0.876 |
|  | Occupational category: others | 1.00 | 0.97–1.03 | 0.876 |
|  | Occupational category: inactive | 0.95 | 0.89–1.00 | 0.068 |
|  | Origin: others | 0.96 | 0.90–1.04 | 0.327 |
| **Dengue and others** | age (years) | 0.99 | 0.96–1.02 | 0.450 |
|  | Neighborhood: Boutillier (Rémire) | 1.02 | 0.96–1.08 | 0.502 |
|  | Neighborhood: PK14 (Macouria) | 1.10 | 0.89–1.35 | 0.391 |
|  | Neighborhood: Terca (Matoury) | 0.97 | 0.85–1.12 | 0.693 |
|  | Sex: male | 0.88 | 0.70–1.10 | 0.260 |
|  | Education: schooled | 0.31 | 0.09–1.02 | 0.054 |
|  | Occupational category: others | 0.97 | 0.29–3.22 | 0.956 |
|  | Occupational category: inactive | 0.00 | - | - |
|  | Origin: others | 0.18 | 0.02–1.44 | 0.105 |
| **Incorrect notion** | age (years) | 0.99 | 0.96–1.02 | 0.450 |
|  | Neighborhood: Boutillier (Rémire) | 1.02 | 0.96–1.08 | 0.502 |
|  | Neighborhood: PK14 (Macouria) | 1.10 | 0.89–1.35 | 0.391 |
|  | Neighborhood: Terca (Matoury) | 0.97 | 0.85–1.12 | 0.693 |
|  | Sex: male | 0.88 | 0.70–1.10 | 0.260 |
|  | Education: schooled | 0.31 | 0.09–1.02 | 0.054 |
|  | Occupational category: others | 0.97 | 0.29–3.22 | 0.956 |
|  | Occupational category: inactive | 0.00 | - | - |
|  | Origin: others | 0.18 | 0.02–1.44 | 0.105 |

- 1. **Variable:** **« Knowledge of dengue symptoms »**

*Table S2.3. Ordinal logistic regression (outcome: knowledge of dengue symptoms)*

Legend: In the multivariable ordinal logistic regression model, only origin was significantly associated with knowledge of dengue symptoms. Participants classified as having an “Other” origin had significantly lower odds of having higher levels of symptom knowledge compared with the reference group (OR = 0.13; 95% CI 0.02–0.70; p = 0.017). No statistically significant associations were found for age, neighborhood, sex, education level, or socio-professional category. These findings suggest that symptom-related knowledge was largely independent of sociodemographic characteristics, with the exception of origin.

| **Variable** | **OR** | **95% CI** | **p-value** |
| --- | --- | --- | --- |
| Age (years) | 1.01 | (0.99–1.03) | 0.324 |
| Neighborhood: Boutillier (Remire) | 1.73 | (0.90–3.31) | 0.100 |
| Neighborhood: PK14 (Macouria) | 1.04 | (0.59–1.86) | 0.883 |
| Neighborhood: Terca (Matoury) | 1.42 | (0.84–2.38) | 0.187 |
| Sex: Male | 1.02 | (0.68–1.55) | 0.916 |
| Education: Schooling | 0.90 | (0.45–1.78) | 0.758 |
| Occupation: Other | 0.81 | (0.34–1.93) | 0.641 |
| Occupation: Inactive | 0.72 | (0.33–1.56) | 0.403 |
| Origin: Other | 0.13 | (0.02–0.70) | 0.017 |

- 1. **Variable : « Attitudes toward dengue »**

*Table S2.4. Multivariate logistic regression (outcome:* Attitudes toward dengue*)*

Legend: In the multivariable logistic regression model, proactive dengue prevention behaviour was not significantly associated with age, neighborhood, education level, occupational category, or origin. Male participants showed a trend toward lower odds of adopting proactive dengue prevention behaviours compared with females; however, this association did not reach statistical significance (OR = 0.59; 95% CI 0.35–1.01; p = 0.053). Overall, no sociodemographic factor was clearly associated with proactive dengue prevention practices.

| **Variable** | **OR** | **95% CI** | **p-value** |
| --- | --- | --- | --- |
| Age | 1.01 | (0.99–1.04) | 0.283 |
| Neighborhood (Boutillier vs Remire) | 0.93 | (0.40–2.15) | 0.864 |
| Neighborhood (PK14 vs Remire) | 1.02 | (0.47–2.22) | 0.958 |
| Neighborhood (Terca vs Remire) | 1.09 | (0.54–2.18) | 0.813 |
| Sex (male vs female) | 0.59 | (0.35–1.01) | 0.053 |
| Education (Schooled vs not) | 0.54 | (0.17–1.70) | 0.295 |
| Occupation (Other vs reference) | 0.67 | (0.19–2.39) | 0.535 |
| Occupation (Inactive vs reference) | 0.54 | (0.17–1.70) | 0.294 |
| Origin (Others vs reference) | 0.54 | (0.06–5.04) | 0.588 |

- 1. **Variable:** **« Individual preventive measures »**

*Table S2.5. Multivariate logistic regression (outcome:* Individual preventive measures*)*

Legend: Overall, no sociodemographic factor was significantly associated with the adoption of protective measures against dengue. Although higher odds were observed among individuals belonging to the “Other” socio-professional category, this association did not reach statistical significance and was accompanied by wide confidence intervals, indicating substantial uncertainty. Age, sex, education level, neighborhood, and origin were not associated with the use of protective measures in this population.

| **Variable** | **OR** | **95% CI** | **p-value** |
| --- | --- | --- | --- |
| Age | 1.00 | (0.97–1.03) | 0.959 |
| Neighborhood: Boutillier (Remire) | 0.59 | (0.23–1.54) | 0.284 |
| Neighborhood: PK14 (Macouria) | 2.91 | (0.64–13.16) | 0.165 |
| Neighborhood: Terca (Matoury) | 1.85 | (0.63–5.44) | 0.262 |
| Male sex | 0.81 | (0.39–1.70) | 0.576 |
| Schooled | 0.95 | (0.24–3.75) | 0.940 |
| Socio-professional category: Other | 7.16 | (0.68–74.88) | 0.100 |
| Socio-professional category: Inactive | 1.28 | (0.33–4.92) | 0.718 |
| Other origin | 0.00 | (0–∞) | 0.987 |

**2.6. Variable:** **« Collective preventive measures »**

*Table S2.6. Multivariate logistic regression (outcome:* Collective preventive measures*)*

Legend: Avoiding mosquito presence was not significantly associated with age, place of residence, education level, socio-professional category, or origin. A non-significant trend toward a higher likelihood of adopting a proactive approach was observed among men compared with women (OR = 1.60; 95% CI 0.96–2.66; p = 0.072). Overall, sociodemographic factors did not strongly influence behaviours aimed at avoiding mosquito presence.

| **Variable** | **OR** | **95% CI** | **p-value** |
| --- | --- | --- | --- |
| Age | 1.01 | (0.99–1.03) | 0.412 |
| District – Boutillier (Remire) | 0.67 | (0.32–1.42) | 0.296 |
| District – PK14 (Macouria) | 1.04 | (0.51–2.11) | 0.924 |
| District – Terca (Matoury) | 1.12 | (0.59–2.11) | 0.733 |
| Male sex | 1.60 | (0.96–2.66) | 0.072 |
| Schooled | 0.49 | (0.18–1.30) | 0.150 |
| CSP – Other | 0.94 | (0.27–3.19) | 0.915 |
| CSP – Inactive | 0.47 | (0.17–1.33) | 0.154 |
| Other origin | 1.14 | (0.20–6.52) | 0.883 |

1. Supplementary Material – Detailed bivariate and multivariate analyses of knowledge, attitudes and practices related to zoonotic diseases (May 2023)

This supplementary material presents the detailed bivariate and multivariate analyses of variables related to knowledge, attitudes and practices on zoonotic diseases, corresponding to Table 11 in the main manuscript.

Taken together, the analyses suggest that sociodemographic characteristics had a limited impact on leptospirosis-related knowledge, attitudes, and practices in this population. While some associations reached statistical significance for specific outcomes, these effects were inconsistent and often accompanied by wide confidence intervals, likely reflecting limited statistical power and small subgroup sizes. Overall, no clear sociodemographic pattern emerged.

**3.1. Variable:** **« Animals: disease transmitters »**

*Table S3.1. Multivariate logistic regression (outcome:* Animals: disease transmitters*)*

Legend: In the multivariate logistic regression model, living in PK14 (Macouria) was associated with lower odds of believing that animals/mosquitoes transmit diseases (OR = 0.36; 95% CI 0.16–0.83; p = 0.017). Participants in the inactive socio-professional category had higher odds of this belief compared to salaried individuals (OR = 3.29; 95% CI 1.11–9.70; p = 0.031). No other sociodemographic factors were significantly associated. The estimates for origin were unstable, likely due to small subgroup sizes.

| **Variable** | **OR** | **95% CI** | **p-value** | |
| --- | --- | --- | --- | --- |
| Age (per year) | 1.00 | 0.97–1.03 | | 0.844 |
| Neighborhood (ref = 30_Pieces) |  |  |  | |
| • Boutillier (Remire) | 0.73 | 0.25–2.13 | 0.561 | |
| • PK14 (Macouria) | 0.36 | 0.16–0.83 | 0.017 | |
| • Terca (Matoury) | 1.39 | 0.54–3.54 | 0.495 | |
| Sex (ref = female) |  |  |  | |
| • Male | 0.95 | 0.48–1.90 | 0.891 | |
| Education (ref = non-schooled) |  |  |  | |
| • Schooled | 0.99 | 0.30–3.25 | 0.981 | |
| Occupational category (ref = salaried) |  |  |  | |
| • Other | 1.67 | 0.50–5.57 | 0.401 | |
| • Inactive | 3.29 | 1.11–9.70 | 0.031 | |
| Origin (ref = France) |  |  |  | |
| • Others | 0.00 | 0–∞ | 0.987 | |

**3.2. Variable:** **« Knowledge of leptospirosis »**

*Table S3.2. Multivariate logistic regression (outcome: Knowledge of leptospirosis)*

Legend: In the multivariate logistic regression model, living in Boutillier (Remire) was significantly associated with higher odds of knowing about leptospirosis compared with the reference neighborhood (OR = 2.76; 95% CI 1.26–6.08; p = 0.012). No other sociodemographic factor was significantly associated with knowledge of leptospirosis in this sample.

| **Variable** | **OR** | **95% CI** | **p-value** |
| --- | --- | --- | --- |
| Age (per year) | 1.02 | 1.00–1.04 | 0.082 |
| Neighborhood (ref = 30_Pieces) |  |  |  |
| • Boutillier (Remire) | 2.76 | 1.26–6.08 | 0.012 |
| • PK14 (Macouria) | 0.66 | 0.35–1.27 | 0.215 |
| • Terca (Matoury) | 0.79 | 0.45–1.38 | 0.406 |
| Sex (ref = female) |  |  |  |
| • Male | 0.73 | 0.47–1.15 | 0.180 |
| Education (ref = non-schooled) |  |  |  |
| • Schooled | 0.72 | 0.32–1.59 | 0.410 |
| Occupational category (ref = salaried) |  |  |  |
| • Other | 0.58 | 0.23–1.51 | 0.267 |
| • Inactive | 0.65 | 0.28–1.49 | 0.311 |
| Origin (ref = France) |  |  |  |
| • Others | 0.56 | 0.11–2.73 | 0.469 |

**3.3. Variable:** **« Knowledge of leptospirosis** **transmission »**

*Table S3.3. Multivariate logistic regression (outcome: Knowledge of leptospirosis transmission)*

Legend:In the multivariable logistic regression model, living in Terca (Matoury) was significantly associated with lower odds of knowing that leptospirosis is transmitted by rats (OR = 0.45; 95% CI 0.24–0.84; p = 0.013). No other sociodemographic factor was significantly associated with this knowledge. The estimate for the “Origin: Others” category was unstable (very wide CI) due to small subgroup sizes.

| **Variable** | **OR** | **95% CI** | **p-value** |
| --- | --- | --- | --- |
| Age (years) | 1.01 | 0.99–1.03 | 0.221 |
| Neighborhood (ref = 30_Pieces) |  |  |  |
| • Boutillier (Remire) | 1.42 | 0.69–2.91 | 0.335 |
| • PK14 (Macouria) | 0.89 | 0.46–1.74 | 0.740 |
| • Terca (Matoury) | 0.45 | 0.24–0.84 | 0.013 |
| Sex (ref = female) |  |  |  |
| • Male | 0.65 | 0.40–1.06 | 0.087 |
| Education (ref = non-schooled) |  |  |  |
| • Schooled | 0.67 | 0.30–1.49 | 0.327 |
| Occupational category (ref = salaried) |  |  |  |
| • Other | 0.64 | 0.23–1.81 | 0.399 |
| • Inactive | 0.69 | 0.28–1.69 | 0.416 |
| Origin (ref = France) |  |  |  |
| • Others | 1,710,732.04 | (0–Inf) | 0.979 |

**3.4. Variable:** **« Knowledge of leptospirosis** **symptoms »**

*Table S3.4. Multivariate logistic regression (outcome: Knowledge of leptospirosis symptoms)*

Legend: In the multivariable ordinal logistic regression model, age was positively associated with better knowledge of leptospirosis symptoms (OR = 1.02; 95% CI 1.00–1.05; p = 0.033). Participants of other origins had lower odds of higher knowledge compared with French origin (OR = 0.19; 95% CI 0.04–0.99; p = 0.049). No other sociodemographic variable was significantly associated.

| **Variable** | **OR** | **95% CI** | **p-value** |
| --- | --- | --- | --- |
| Age (years) | 1.02 | 1.00–1.05 | 0.033 |
| Neighborhood (ref = 30_Pieces) |  |  |  |
| • Boutillier (Remire) | 1.33 | 0.65–2.72 | 0.430 |
| • PK14 (Macouria) | 0.78 | 0.38–1.60 | 0.496 |
| • Terca (Matoury) | 0.53 | 0.28–1.03 | 0.060 |
| Sex (ref = female) |  |  |  |
| • Male | 1.12 | 0.69–1.83 | 0.634 |
| Education (ref = non-schooled) |  |  |  |
| • Schooled | 0.75 | 0.35–1.62 | 0.466 |
| Occupational category (ref = salaried) |  |  |  |
| • Other | 1.00 | 0.30–3.33 | 0.995 |
| • Inactive | 1.51 | 0.53–4.29 | 0.441 |
| Origin (ref = France) |  |  |  |
| • Others | 0.19 | 0.04–0.99 | 0.049 |

**3.5. Variable:** **« Attitudes toward leptospirosis** **»**

*Table S3.5. Multivariate logistic regression (outcome: Attitudes toward leptospirosis)*

Legend: In the multivariable logistic regression model, older age was associated with slightly lower odds of having a proactive attitude toward leptospirosis symptoms (OR = 0.98; 95% CI 0.96–1.00; p = 0.021). Living in Boutillier (Remire) was associated with lower odds of proactive attitude compared with 30_Pieces (OR = 0.43; 95% CI 0.19–0.95; p = 0.036). No other sociodemographic factors were significantly associated. There was a non-significant trend for men to have higher odds of proactive attitude compared with women (OR = 1.50; 95% CI 0.95–2.37; p = 0.081).

| **Variable** | **OR** | **95% CI** | **p-value** |
| --- | --- | --- | --- |
| Age (years) | 0.98 | 0.96–1.00 | 0.021 |
| Neighborhood (ref = 30_Pieces) |  |  |  |
| • Boutillier (Remire) | 0.43 | 0.19–0.95 | 0.036 |
| • PK14 (Macouria) | 1.58 | 0.82–3.03 | 0.169 |
| • Terca (Matoury) | 1.54 | 0.87–2.72 | 0.137 |
| Sex (ref = female) |  |  |  |
| • Male | 1.50 | 0.95–2.37 | 0.081 |
| Education (ref = non-schooled) |  |  |  |
| • Schooled | 1.16 | 0.52–2.59 | 0.718 |
| Occupational category (ref = salaried) |  |  |  |
| • Other | 1.50 | 0.58–3.91 | 0.404 |
| • Inactive | 0.96 | 0.42–2.22 | 0.933 |
| Origin (ref = France) |  |  |  |
| • Others | 0.62 | 0.11–3.47 | 0.588 |

**3.6. Variable:** **« Individual preventive measures** **»**

*Table S3.6. Multivariate logistic regression (outcome: Individual preventive measures)*

Legend: In the multivariable logistic regression model, no sociodemographic factor was significantly associated with proactive actions to avoid mosquito presence. There was a non-significant tendency for men to be more likely to adopt proactive measures (OR = 1.72; 95% CI 0.85–3.47; p = 0.130) and for individuals in the “other” occupational category to show higher odds (OR = 3.41; 95% CI 0.85–13.78; p = 0.085), but these associations were not statistically significant and confidence intervals were wide.

| **Variable** | **OR** | **95% CI** | **p-value** |
| --- | --- | --- | --- |
| Age (per year) | 0.99 | 0.96–1.02 | 0.380 |
| Neighborhood (ref = 30_Pieces) |  |  |  |
| • Boutillier (Remire) | 0.57 | 0.23–1.41 | 0.221 |
| • PK14 (Macouria) | 1.80 | 0.58–5.60 | 0.309 |
| • Terca (Matoury) | 1.10 | 0.48–2.56 | 0.819 |
| Sex (ref = female) |  |  |  |
| • Male | 1.72 | 0.85–3.47 | 0.130 |
| Education (ref = not schooled) |  |  |  |
| • Schooled | 0.93 | 0.30–2.87 | 0.903 |
| Occupational category (ref = employed) |  |  |  |
| • Other | 3.41 | 0.85–13.78 | 0.085 |
| • Inactive | 2.20 | 0.76–6.35 | 0.145 |
| Origin (ref = France) |  |  |  |
| • Others | 0.00 | 0–Inf | 0.987 |

**3.7. Variable:** **« Collective preventive measures** **»**

*Table S3.7. Multivariate logistic regression (outcome: Collective preventive measures)*

Legend: In the multivariable logistic regression model adjusted for age, neighborhood, sex, education, occupational category and origin, men were significantly more likely to report using preventive measures against leptospirosis (OR = 2.06; 95% CI 1.17–3.64; p = 0.013). No other sociodemographic variable was significantly associated with the adoption of preventive measures.

| **Variable** | **OR** | **95% CI** | **p-value** |
| --- | --- | --- | --- |
| Age (per year) | 0.99 | 0.96–1.01 | 0.284 |
| Neighborhood (ref = 30_Pieces) |  |  |  |
| • Boutillier (Remire) | 1.76 | 0.68–4.56 | 0.247 |
| • PK14 (Macouria) | 1.28 | 0.59–2.78 | 0.537 |
| • Terca (Matoury) | 1.23 | 0.63–2.39 | 0.547 |
| Sex (ref = female) |  |  |  |
| • Male | 2.06 | 1.17–3.64 | 0.013 |
| Education (ref = non-schooled) |  |  |  |
| • Schooled | 0.54 | 0.20–1.48 | 0.230 |
| Occupational category (ref = salaried) |  |  |  |
| • Other | 0.44 | 0.12–1.55 | 0.201 |
| • Inactive | 0.55 | 0.17–1.76 | 0.317 |
| Origin (ref = France) |  |  |  |
| • Others | 1.87 | 0.32–10.95 | 0.489 |

4. Supplementary Material – Detailed analyses of water access and water treatment practices among residents of informal settlements (May 2023)

This supplementary material presents detailed analyses of variables related to water access and water treatment practices, corresponding to Table 4 in the main manuscript.

Taken together, the results suggest that water treatment practices were only partially explained by sociodemographic factors. The observed associations were mainly related to neighborhood of residence, whereas other individual-level characteristics showed no robust or systematic influence. Some estimates were imprecise, with wide confidence intervals, suggesting limited statistical power for certain subgroups.

**4.1. Variable: *«*Water treatment *»***

*Table S4.1. Multivariate logistic regression (outcome: Water treatment)*

Legend : In the multivariable logistic regression model adjusted for age, neighborhood, sex, education, occupational category and origin, living in Boutillier (Remire) and PK14 (Macouria) was significantly associated with a higher likelihood of treating water. No other sociodemographic variable was significantly associated with treating water.

| **Variable** | **OR** | **95% CI** | **p-value** |
| --- | --- | --- | --- |
| Age (per year) | 1.02 | 1.00–1.04 | 0.067 |
| Neighborhood (ref = 30_Pieces) |  |  |  |
| • Boutillier (Remire) | 2.18 | 1.05–4.69 | 0.040 |
| • PK14 (Macouria) | 4.38 | 2.14–9.62 | <0.001 |
| • Terca (Matoury) | 0.83 | 0.47–1.46 | 0.528 |
| Sex (ref = female) |  |  |  |
| • Male | 0.95 | 0.60–1.50 | 0.813 |
| Education (ref = non-schooled) |  |  |  |
| • Schooled | 0.73 | 0.31–1.63 | 0.447 |
| Occupational category (ref = salaried) |  |  |  |
| • Others | 0.50 | 0.19–1.32 | 0.166 |
| • Inactive | 0.55 | 0.23–1.27 | 0.164 |
| Origin (ref = France) |  |  |  |
| • Others | 0.97 | 0.19–5.33 | 0.965 |

**4.2. Variable: *«*Treatment with disinfectant products *»***

*Table S4.2. Multivariate logistic regression (outcome:* Treatment with disinfectant products*)*

Legend : Multinomial logistic regression model adjusted for age, neighborhood, sex, education, occupational category and origin. The reference category is not treating water (tt_pas). The table shows factors associated with choosing another treatment (Autre_tt) compared with not treating water.

| **Variable** | **OR** | **95% CI** | **p-value** |
| --- | --- | --- | --- |
| Age (per year) | 0.98 | 0.93–1.03 | 0.408 |
| Neighborhood (ref = 30_Pieces) |  |  |  |
| • Boutillier (Remire) | 0.24 | 0.03–1.99 | 0.185 |
| • PK14 (Macouria) | 0.12 | 0.01–1.03 | 0.054 |
| • Terca (Matoury) | 0.40 | 0.07–2.15 | 0.284 |
| Sex (ref = female) |  |  |  |
| • Male | 0.34 | 0.10–1.24 | 0.102 |
| Education (ref = non-schooled) |  |  |  |
| • Schooled | 0.70 | 0.12–3.94 | 0.685 |
| Occupational category (ref = salaried) |  |  |  |
| • Others | 0.72 | 0.02–26.7 | 0.860 |
| • Inactive | 3.02 | 0.16–56.5 | 0.459 |
| Origin (ref = France) |  |  |  |
| • Others | 0.02 | 0.00–0.39 | 0.011 |

**4.3. Variable: « Water treatment time before consumption »**

*Table S4.3. Multivariate logistic regression (outcome:* Water treatment time before consumption*)*

Legend : In the multivariable logistic regression model adjusted for age, neighborhood, sex, education, occupational category and origin, living in Boutillier (Remire) was significantly associated with a higher likelihood of consuming water for more than 30 minutes (good practice). No other sociodemographic variable was significantly associated with this behavior.

| **Variable** | **OR** | **95% CI** | **p-value** |
| --- | --- | --- | --- |
| Age (per year) | 1.00 | 0.96–1.03 | 0.912 |
| Neighborhood (ref = 30_Pieces) |  |  |  |
| • Boutillier (Remire) | 2.99 | 0.99–8.90 | 0.048 |
| • PK14 (Macouria) | 0.86 | 0.27–2.46 | 0.781 |
| • Terca (Matoury) | 0.96 | 0.28–3.05 | 0.949 |
| Sex (ref = female) |  |  |  |
| • Male | 1.12 | 0.49–2.50 | 0.787 |
| Education (ref = non-schooled) |  |  |  |
| • Schooled | 1.69 | 0.47–8.29 | 0.459 |
| Occupational category (ref = salaried) |  |  |  |
| • Others | 1.28 | 0.21–10.72 | 0.799 |
| • Inactive | 1.38 | 0.30–10.00 | 0.706 |
| Origin (ref = France) |  |  |  |
| • Others | 0.42 | 0.03–10.21 | 0.513 |

**4.4. Variable: « Water storage time after treatment »**

*Table S4.4. Multivariate logistic regression (outcome:* Water storage time after treatment*)*

Legend: In the multivariable logistic regression model adjusted for age, neighborhood, sex, education, occupational category and origin, no sociodemographic factor was significantly associated with correct water conservation time (≤ 2 days). The reference category is mauvaise_pratique (conservation > 2 days or unknown).

| **Variable** | **OR** | **95% CI** | **p-value** |
| --- | --- | --- | --- |
| Age (per year) | 0.99 | 0.97–1.02 | 0.680 |
| Neighborhood (ref = 30_Pieces) |  |  |  |
| • Boutillier (Remire) | 0.48 | 0.17–1.31 | 0.154 |
| • PK14 (Macouria) | 0.86 | 0.38–1.97 | 0.715 |
| • Terca (Matoury) | 0.46 | 0.18–1.15 | 0.099 |
| Sex (ref = female) |  |  |  |
| • Male | 0.95 | 0.49–1.84 | 0.871 |
| Education (ref = non-schooled) |  |  |  |
| • Schooled | 1.13 | 0.41–3.18 | 0.814 |
| Occupational category (ref = salaried) |  |  |  |
| • Others | 0.71 | 0.16–3.17 | 0.657 |
| • Inactive | 0.93 | 0.24–3.49 | 0.916 |
| Origin (ref = France) |  |  |  |
| • Others | 0.31 | 0.01–3.69 | 0.365 |

1. Supplementary Material – Detailed analyses of water collection, transport, and storage practices among residents of informal settlements (May 2023)

This supplementary material presents detailed analyses of variables related to water collection, transport, and storage practices, corresponding to Table 6 in the main manuscript.

Overall, the multivariate analyses showed that neighborhood of residence was the main factor associated with water collection, transport, and storage practices. Participants from Terca (Matoury) and Boutillier (Remire) exhibited significant differences in certain practices, such as type of container used, container closure during storage, and water storage at home. In contrast, no consistent associations were observed for age, sex, education level, occupational category, or origin, and confidence intervals were often wide, indicating uncertainty for these variables. Thus, sociodemographic characteristics had limited explanatory power for these behaviors in this population.

**5.1. Variable: *«*Type of container used *»***

*Table S5.1. Multivariate logistic regression (outcome:* Type of container used*)*

Legend: In the multivariable logistic regression model adjusted for age, neighborhood, sex, education, occupational category and origin, living in Terca (Matoury) was significantly associated with correct water treatment type (good practice). The reference category is mauvaise_pratique (type 0). Good practice corresponds to types 1 and 2.

| **Variable** | **OR** | **95% CI** | **p-value** |
| --- | --- | --- | --- |
| Age (per year) | 0.99 | 0.97–1.01 | 0.420 |
| Neighborhood (ref = 30_Pieces) |  |  |  |
| • Boutillier (Remire) | 0.92 | 0.45–1.91 | 0.820 |
| • PK14 (Macouria) | 1.06 | 0.55–2.06 | 0.860 |
| • Terca (Matoury) | 2.37 | 1.28–4.50 | 0.007 |
| Sex (ref = female) |  |  |  |
| • Male | 0.84 | 0.53–1.35 | 0.476 |
| Education (ref = non-schooled) |  |  |  |
| • Schooled | 0.94 | 0.40–2.09 | 0.874 |
| Occupational category (ref = salaried) |  |  |  |
| • Others | 0.55 | 0.17–1.59 | 0.289 |
| • Inactive | 0.66 | 0.23–1.67 | 0.399 |
| Origin (ref = France) |  |  |  |
| • Others | 1.42 | 0.19–7.48 | 0.696 |

**5.2. Variable: *«*Frequency of container washing *»***

*Table S5.2. Multivariate logistic regression (outcome:* Frequency of container washing*)*

Legend: In the multivariable logistic regression model assessing factors associated with good frequency of container cleaning, no sociodemographic variable was significantly associated with good practice. The odds ratios for neighborhood, sex, education, occupational category, and origin were not statistically significant, and confidence intervals were wide, indicating uncertainty and lack of clear evidence for association in this sample. Although the odds ratio for Boutillier (Remire) suggested higher odds of good practice compared with 30_Pieces (OR = 2.14), this association was not statistically significant (p = 0.478).

| **Variable** | **OR** | **95% CI** | **p-value** |
| --- | --- | --- | --- |
| Age (per year) | 1.01 | 0.97–1.06 | 0.519 |
| Neighborhood (ref = 30_Pieces) |  |  |  |
| • Boutillier (Remire) | 2.14 | 0.38–40.02 | 0.478 |
| • PK14 (Macouria) | 0.61 | 0.18–2.38 | 0.437 |
| • Terca (Matoury) | 0.94 | 0.28–3.67 | 0.925 |
| Sex (ref = female) |  |  |  |
| • Male | 0.47 | 0.18–1.23 | 0.124 |
| Education (ref = non-schooled) |  |  |  |
| • Schooled | 0.65 | 0.03–3.84 | 0.690 |
| Occupational category (ref = salaried) |  |  |  |
| • Others | 2.35 | 0.26–21.57 | 0.419 |
| • Inactive | 1.17 | 0.17–4.92 | 0.849 |
| Origin (ref = France) |  |  |  |
| • Others | 2.52 | 0.11–22.22 | 0.456 |

**5.3. Variable: *«*Washing of storage containers *»***

*Table S5.3. Multivariate logistic regression (outcome:* Washing of storage containers*)*

Legend: In the multivariable logistic regression model assessing factors associated with correct washing procedure (good practice vs poor practice), no sociodemographic variable was significantly associated. The odds ratios for neighborhood, sex, education, occupational category, and origin were not statistically significant, and confidence intervals were wide, suggesting uncertainty and no clear evidence of association in this sample. The estimate for education is unstable (OR = 0 and extremely wide confidence interval), indicating potential separation or sparse data in this category; therefore, this result should be interpreted with caution.

| **Variable** | **OR** | **95% CI** | **p-value** |
| --- | --- | --- | --- |
| Age (per year) | 1.03 | 0.98–1.08 | 0.305 |
| Neighborhood (ref = 30_Pieces) |  |  |  |
| • Boutillier (Remire) | 0.66 | 0.15–4.63 | 0.619 |
| • PK14 (Macouria) | 0.87 | 0.19–6.15 | 0.869 |
| • Terca (Matoury) | 0.58 | 0.15–2.41 | 0.431 |
| Sex (ref = female) |  |  |  |
| • Male | 0.48 | 0.16–1.37 | 0.171 |
| Education (ref = non-schooled) |  |  |  |
| • Schooled | 0.00 | NA–7.96×10²² | 0.989 |
| Occupational category (ref = salaried) |  |  |  |
| • Others | 1.88 | 0.21–17.16 | 0.548 |
| • Inactive | 1.30 | 0.18–5.69 | 0.753 |
| Origin (ref = France) |  |  |  |
| • Others | 2.71 | 0.12–25.31 | 0.431 |

**5.4. Variable: *«*Closing of containers during transport *»***

*Table S5.4. Multivariate logistic regression (outcome:* Closing of containers during transport)

Legend: In the multivariable logistic regression model assessing factors associated with covering the container during transport, no sociodemographic variable was significantly associated with good practice. The odds ratios for neighborhood, sex, education, occupational category, and origin were not statistically significant and confidence intervals were wide, indicating uncertainty and no clear evidence of association. The estimate for origin is unstable (OR = 0 with an extremely wide confidence interval), suggesting sparse data or separation in this category; therefore, this result should be interpreted with caution.

| **Variable** | **OR** | **95% CI** | **p-value** |
| --- | --- | --- | --- |
| Age (per year) | 0.99 | 0.95–1.03 | 0.711 |
| Neighborhood (ref = 30_Pieces) |  |  |  |
| • Boutillier (Remire) | 1.72 | 0.45–11.28 | 0.488 |
| • PK14 (Macouria) | 1.48 | 0.45–6.72 | 0.556 |
| • Terca (Matoury) | 1.51 | 0.50–5.36 | 0.487 |
| Sex (ref = female) |  |  |  |
| • Male | 1.17 | 0.48–2.97 | 0.733 |
| Education (ref = non-schooled) |  |  |  |
| • Schooled | 0.73 | 0.10–3.10 | 0.704 |
| Occupational category (ref = salaried) |  |  |  |
| • Others | 0.70 | 0.03–5.97 | 0.761 |
| • Inactive | 0.59 | 0.03–3.35 | 0.625 |
| Origin (ref = France) |  |  |  |
| • Others | 0.00 | NA–1.22×10²⁶ | 0.989 |

**5.5. Variable: *«*Closing of containers during storage *»***

*Table S5.5. Multivariate logistic regression (outcome:* Closing of containers during storage)

Legend: In the multivariable logistic regression model assessing factors associated with covering the container during storage, only neighborhood was significantly associated.
Participants from Terca (Matoury) had significantly lower odds of reporting good practice compared to those from 30_Pieces (OR = 0.11; 95% CI 0.02–0.40; p = 0.002). No other sociodemographic factor (age, sex, education, occupational category, or origin) was significantly associated with covering the container during storage.

| **Variable** | **OR** | **95% CI** | **p-value** |
| --- | --- | --- | --- |
| Age (per year) | 0.99 | 0.96–1.03 | 0.624 |
| Neighborhood (ref = 30_Pieces) |  |  |  |
| • Boutillier (Remire) | 0.35 | 0.06–2.79 | 0.269 |
| • PK14 (Macouria) | 0.94 | 0.12–19.30 | 0.955 |
| • Terca (Matoury) | 0.11 | 0.02–0.40 | 0.002 |
| Sex (ref = female) |  |  |  |
| • Male | 2.17 | 0.78–7.12 | 0.163 |
| Education (ref = non-schooled) |  |  |  |
| • Schooled | 0.29 | 0.01–1.84 | 0.269 |
| Occupational category (ref = salaried) |  |  |  |
| • Others | 3.56 | 0.64–27.66 | 0.165 |
| • Inactive | 1.54 | 0.41–5.09 | 0.498 |
| Origin (ref = France) |  |  |  |
| • Others | 2.71 | 0.32–17.61 | 0.310 |

**5.6. Variable: « Water storage at home »**

*Table S5.6. Multivariate logistic regression (outcome:* Water storage at home)

Legend: In the multivariable logistic regression model assessing factors associated with adequate water storage practice, only neighborhood was significantly associated. Participants living in Boutillier (Remire) had higher odds of reporting adequate water storage practices compared to those living in 30_Pieces (OR = 3.44; 95% CI 1.61–7.30; p = 0.001). No other sociodemographic factor (age, sex, education, occupational category, or origin) was significantly associated with adequate storage practice.

| **Variable** | **OR** | **95% CI** | **p-value** |
| --- | --- | --- | --- |
| Age (per year) | 1.00 | 0.98–1.03 | 0.666 |
| Neighborhood (ref = 30_Pieces) |  |  |  |
| • Boutillier (Remire) | 3.44 | 1.61–7.30 | 0.001 |
| • PK14 (Macouria) | 0.72 | 0.29–1.65 | 0.463 |
| • Terca (Matoury) | 1.64 | 0.86–3.12 | 0.130 |
| Sex (ref = female) |  |  |  |
| • Male | 0.99 | 0.58–1.67 | 0.967 |
| Education (ref = non-schooled) |  |  |  |
| • Schooled | 1.06 | 0.44–2.73 | 0.902 |
| Occupational category (ref = salaried) |  |  |  |
| • Others | 1.26 | 0.46–3.68 | 0.658 |
| • Inactive | 0.83 | 0.34–2.19 | 0.686 |
| Origin (ref = France) |  |  |  |
| • Others | 0.95 | 0.19–7.10 | 0.957 |

1. [↑](#footnote-ref-1)
2. [↑](#footnote-ref-2)
3. [↑](#footnote-ref-3)
